# Deciphering the role of apoptosis signature on the immune dynamics and therapeutic prognosis in breast cancer: Implication for immunotherapy

**DOI:** 10.3389/fgene.2024.1332935

**Published:** 2024-05-02

**Authors:** Yunfang Yu, Xueyuan Jia, Sunyu Chen, Zijia Lai, Heran Deng, Yuqian Mo, Xinxin Xie, Zehua Wang, Ruichong Lin, Wenhao Ouyang, Herui Yao, Jiannan Wu

**Affiliations:** ^1^ Faculty of Medicine, Macau University of Science and Technology, Taipa, Macao, China; ^2^ Guangdong Provincial Key Laboratory of Malignant Tumor Epigenetics and Gene Regulation, Phase I Clinical Trial Cent, Breast Tumor Center, Sun Yat-sen Memorial Hospital, Sun Yat-sen University, Guangzhou, China; ^3^ School of Clinical Medicine, Guangdong Medical University, Zhanjiang, China; ^4^ Division of Science and Technology, Beijing Normal University-Hong Kong Baptist University United International College, Hong Kong Baptist University, Zhuhai, China; ^5^ School of Computer Engineering, Guangzhou Huali College, Guangzhou, China; ^6^ Faculty of Innovation Engineering, Macau University of Science and Technology, Taipa, Macao, China

**Keywords:** apoptosis, oncogenic processes, prognostic model, breast cancer, anti-PD-L1 therapy

## Abstract

**Background:** In breast cancer oncogenesis, the precise role of cell apoptosis holds untapped potential for prognostic and therapeutic insights. Thus, it is important to develop a model predicated for breast cancer patients’ prognosis and immunotherapy response based on apoptosis-related signature.

**Methods:** Our approach involved leveraging a training dataset from The Cancer Genome Atlas (TCGA) to construct an apoptosis-related gene prognostic model. The model’s validity was then tested across several cohorts, including METABRIC, Sun Yat-sen Memorial Hospital Sun Yat-sen University (SYSMH), and IMvigor210, to ensure its applicability and robustness across different patient demographics and treatment scenarios. Furthermore, we utilized Quantitative Polymerase Chain Reaction (qPCR) analysis to explore the expression patterns of these model genes in breast cancer cell lines compared to immortalized mammary epithelial cell lines, aiming to confirm their differential expression and underline their significance in the context of breast cancer.

**Results:** Through the development and validation of our prognostic model based on seven apoptosis-related genes, we have demonstrated its substantial predictive power for the survival outcomes of breast cancer patients. The model effectively stratified patients into high and low-risk categories, with high-risk patients showing significantly poorer overall survival in the training cohort and across all validation cohorts. Importantly, qPCR analysis confirmed that the genes constituting our model indeed exhibit differential expression in breast cancer cell lines when contrasted with immortalized mammary epithelial cell lines.

**Conclusion:** Our study establishes a groundbreaking prognostic model using apoptosis-related genes to enhance the precision of breast cancer prognosis and treatment, particularly in predicting immunotherapy response.

## Introduction

Given the high incidence and mortality associated with breast cancer, it remains a critical health concern for women. Despite advances in diverse therapeutic modalities (Harbeck and Gnant, 2017; Sung et al., 2021), conventional prognostic indicators such as patient age, lymphovascular involvement, histological subtype, pathological grading, and radiographic features have limitations in accurately predicting clinical outcomes (Saini et al., 2019; Yu et al., 2020a).

Apoptosis, a highly conserved form of programmed cell death, plays a crucial role in physiological development and maintaining tissue homeostasis (Mohammad et al., 2015). Its regulatory pathways ensure the timely elimination of damaged or unnecessary cells, thus preventing potential malfunctions or the onset of diseases. However, in the context of oncogenesis, particularly within breast cancer cells, the mechanisms that control apoptosis become significantly disrupted. This dysregulation allows cancer cells to evade normal cell death processes, leading to their uncontrolled growth, resistance to conventional therapies, and an increased likelihood of disease recurrence (Carneiro and El-Deiry, 2020). The evasion of apoptosis by cancer cells is a hallmark of cancer that facilitates tumor progression and metastasis by allowing the survival of cells that would otherwise be eliminated by programmed cell death mechanisms. The alteration in apoptotic pathways in cancer cells can be attributed to various genetic and epigenetic changes, including mutations in genes that encode apoptotic regulators and alterations in the expression of microRNAs that target components of the apoptotic machinery. These changes can lead to the overexpression of anti-apoptotic proteins, such as Bcl-2, or the downregulation of pro-apoptotic factors, thereby tipping the balance in favor of cell survival (Pandey et al., 2020; Qu et al., 2020; Kandhavelu et al., 2024).

Given the important role of apoptosis in controlling cancer development. Targeting the dysregulated apoptotic pathways in breast cancer represents a promising therapeutic approach. This strategy involves the development of agents that can specifically induce apoptosis in cancer cells without harming normal tissues, offering a more targeted and potentially less toxic treatment option compared to traditional chemotherapy and radiation therapy (Zhang et al., 2017). Such approaches include the use of BH3 mimetics that inhibit Bcl-2 proteins, activating death receptors through agonistic antibodies, or directly activating caspases with synthetic peptides (Diepstraten et al., 2022).

The tumor microenvironment (TME), a complex assembly of non-malignant cellular components juxtaposed with tumor cells, modulates apoptotic pathways and consequently reconditions cancer cell behavior (Wong, 2011; Deepak et al., 2020; Li et al., 2020). Therapeutic interventions designed to either potentiate apoptosis or counter apoptosis-resistance mechanisms are considered promising approaches (Lv et al., 2011). Cancer cell apoptosis can affect the tumor microenvironment factors to promote the immune cells’ function. A recent study reported that the apoptosis pathway of cell mitochondria can significantly improve the killing of cancer cells by NK cells and is more sensitive to the NK-mediated killing (Pan et al., 2022).

While the significance of apoptosis in the development of tumors is widely recognized, the potential of genes associated with apoptosis to serve as reliable markers for prognosis or immunotherapy for breast cancer has not been sufficiently proven. This study aimed to develop a signature based on apoptosis-related genes, which would not only be predictive of breast cancer prognosis but also provide insights into the tumor microenvironment and forecast responses to immunotherapy, which can enhance the precision of cancer prognosis and open new sight for targeted therapies, ultimately contributing to more personalized and effective treatment strategies for patients.

## Materials and methods

### Patients and study design

In this study, the multi-dimensional genomic and clinical datasets were obtained from several established platforms. Specifically, The Cancer Genome Atlas (TCGA) contributed a cohort of 1,089 breast cancer patients, sequenced on the Illumina-HiSeq platform. The Molecular Taxonomy of Breast Cancer International Consortium (METABRIC) provided data for 1,903 patients, obtained via the Illumina HT-12 v3 platform. Additionally, 74 breast cancer patients were included from the Sun Yat-sen Memorial Hospital, Sun Yat-sen University (SYSMH) cohort, which utilized the DNBSEQ-T7RS (MGI) sequencing technology. Moreover, the IMvigor210 clinical trial dataset, comprising 348 bladder cancer patients treated with atezolizumab (a PD-L1 inhibitor), was also incorporated for specific analyses.

For methodological rigor, the TCGA dataset was designated as the discovery or training set, whereas METABRIC, SYSMH, and IMvigor210 datasets (identified by clinical trial numbers NCT02951767/NCT02108652) were used for external validation purposes. The data were accessed from reputable repositories: TCGA database (https://portal.gdc.cancer.gov/repository), cBioPortal for METABRIC data (http://www.cbioportal.org/), and the IMvigor210 Core Biologies package for IMvigor210 clinical profiles.

The gene panel for apoptotic regulation was meticulously curated from the Molecular Signatures Database V7.0. This database is comprehensive, featuring 161 genes either directly participating in, or indirectly modulating, apoptotic pathways. Such an exhaustive list enables a more nuanced understanding of apoptotic mechanisms in the context of breast cancer, thereby enhancing the study’s potential impact.

### Constructing an anticipating signature of apoptosis-associated gene

The Wilcoxon test was employed to profile genes manifesting significant differential expression between tumor and adjacent non-tumorous tissue, using stringent selection criteria: an absolute log2 fold-change (FC) exceeding 1 and a false discovery rate (FDR)-corrected *p*-value below 0.05.

Further, univariate Cox proportional hazards regression was conducted to ascertain apoptosis-related genes demonstrating both differential expression and significant prognostic value. This was followed by stepwise regression to refine the portfolio of candidate genes for the construction of a predictive prognostic model. The risk score metric for each patient was derived by combining the expression status of individual genes and their corresponding regression coefficients, according to a linear combination model.

$$Risk\;score=\sum_{i=1}^{n}\text{coefficient}\left(i\right)\;*expression\left(i\right)$$

In this model, coefficient(i) and expression(i) symbolize the survival-associated coefficient for gene i and gene i’s expression level, respectively. Utilizing an optimal threshold, patients were stratified into high-risk or low-risk groups. Analysis of overall survival (OS) discrepancies between these risk groups was performed by leveraging the R packages “survival” and “survminer.” The algorithm’s predictive accuracy was validated through time-dependent receiver operating characteristic (ROC) curve analysis, utilizing the “survivalROC” R package.

### The investigation of tumor immune microenvironment

To derive a panoramic understanding of the interplay between risk stratification and immune cell infiltration, we evaluated the distribution of 22 immune cell subtypes in the training cohort using the CIBERSORT analytic tool (http://cibersort.stanford.edu/) (Newman et al., 2015). Differential proportions of immune cell infiltrates between high-risk and low-risk groups were elucidated using the Wilcoxon test.

The Kaplan-Meier method was applied to probe the association of immune cell infiltration levels with overall survival (OS) in the breast cancer cohort. Additionally, we employed the ESTIMATE algorithm, facilitated through the R package “estimate,” to dissect the cellular composition of the tumor microenvironment (TME), focusing on the proportion of immune and stromal cells, alongside the cumulative ESTIMATE score within the TCGA cohort (Yoshihara et al., 2013).

Further, we leveraged the single-sample gene set enrichment analysis (ssGSEA) methodology, facilitated via the “gsva” R package, to gauge the infiltration levels of 16 immune cell types and the functional status of 7 immune-related pathways (Hänzelmann et al., 2013). This comprehensive assessment aimed to elucidate the functional landscape of the TME with respect to immune infiltration.

### Functional enrichment analysis

For enrichment analysis, we employed the “clusterProfiler” package in R (Wu et al., 2021) to execute Gene Ontology (GO) and Kyoto Encyclopedia of Genes and Genomes (KEGG) pathway evaluations. We considered an enrichment to be statistically significant if the false discovery rate (FDR) was less than 0.05, serving as a robust threshold for discerning substantial biological implications.

### Epigenomic and genomic difference analysis

We generated a dataset of somatic mutations, formatted in Mutation Annotation Format (MAF), and proceeded to conduct an in-depth analysis using the ‘maftools’ package in R.

### The predictive nomogram

Utilizing the ‘rms’ package in R, we generated line plots and calibration curves to visualize model performance. To scrutinize the independent prognostic potential of risk scores, clinical parameters, and immune cell infiltration levels for overall survival (OS), both univariate and multivariate Cox proportional hazards analyses were executed. Subsequently, we constructed a prognostic nomogram that integrated the results from multivariate Cox regression, risk stratification models, clinical variables, and immune cell markers. This nomogram aimed to provide quantitative prognostic estimates for OS at 3, 5, and 10-year intervals in breast cancer patients.

### Cells culture

The cell lines employed in this study were acquired from the American Type Culture Collection (ATCC) and cultivated under conventional laboratory conditions. In particular, the breast cancer cell lines, namely SK-BR-3 and MDA-MB-231, were propagated in Dulbecco’s Modified Eagle Medium (DMEM) (Gibco, New York, United States). Meanwhile, the BT-474 and MCF-7 cell lines were maintained in RPMI-1640 medium (Gibco, New York, United States). Both media were enhanced with 10% fetal bovine serum (FBS) (Gibco, New York, United States) and a 1% solution of penicillin-streptomycin (Gibco, New York, United States). Notably, the non-transformed mammary epithelial cell line MCF-10A was sustained in a specialized media formulation (Procell, Wuhan, China). All cell lines were cultured in a humidified incubator at 37 °C with an atmosphere of 5% carbon dioxide (CO_2_).

### RNA isolation, cDNA synthesis, and qPCR analysis

Total RNA extracted from MCF-10A, BT-474, MCF-7, SK-BR-3, and MDA-MB-231 cells, using the TRIzol reagent (Thermo Fisher Scientific, USA), was reverse transcribed using the HiScript III First Strand cDNA Synthesis Kit (Vazyme, Nanjing, China). This was followed by quantitative real-time PCR (qPCR) analysis utilizing the Applied Biosystems’ QuantStudio TMDx platform in combination with the ChamQ Universal SYBR qPCR Master Mix kit (Vazyme, Nanjing, China).

The reaction conditions consisted of an initial polymerase activation step at 95°C for 30 s and 40 subsequent cycles comprising denaturation at 95°C for 5 s and annealing/extension at 60°C for 30 s. The 2^−ΔΔCT^ method was employed for calculating the relative expression levels, standardized against a housekeeping gene.

### Statistical analysis

To identify differentially expressed genes (DEGs) between breast cancer tissues and adjacent normal tissues, we employed the Wilcoxon test. Genes with prognostic significance for overall survival (OS) were then isolated through univariate Cox regression analysis. Patient stratification into high-risk and low-risk cohorts was performed based on an optimal cut-off value, determined using the ‘survminer’ R package.

The Kaplan-Meier survival analysis, corroborated by a log-rank test, was utilized to discern differences in OS between risk groups. Furthermore, receiver operating characteristic (ROC) analysis was conducted to assess the predictive robustness of the identified features. The area under the ROC curve (AUC) was calculated to quantify sensitivity and specificity. All statistical procedures were executed in R version 4.0.0, employing a significance level of *p* < 0.05 for hypothesis testing.

## Results

**FIGURE 1:**
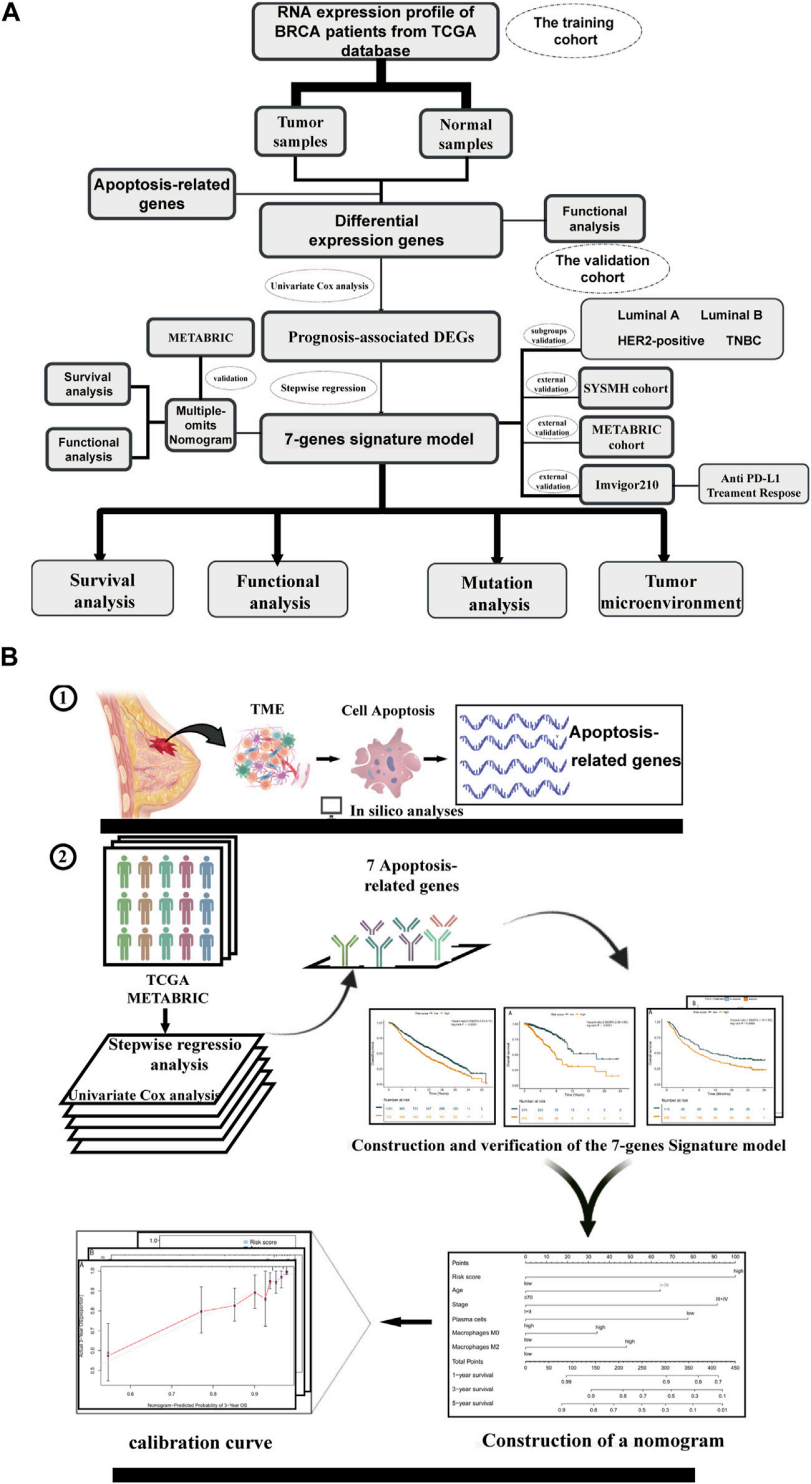
Overview of study flow chart Schematic flowchart of our study on apoptosis-associated prognostic signatures of breast cancer. **(A)** The workflow chart of this study. **(B)** The specific research plan of this study.

In the present investigation, we undertook a comprehensive analysis of 3,066 individual patients, leveraging both mRNA expression profiles and genomic data in strict adherence to the TRIPOD guidelines (Collins et al., 2015). The patient cohort, delineated in Figure 1, encompassed 1,089 subjects from the TCGA-BRCA dataset and an additional 1,903 from the METABRIC cohort. Comprehensive clinical attributes of the study population have been collated and are available for perusal in Supplementary Table S1.

### Apoptosis-related prognostic DEGs in the TCGA cohort

**FIGURE 2:**
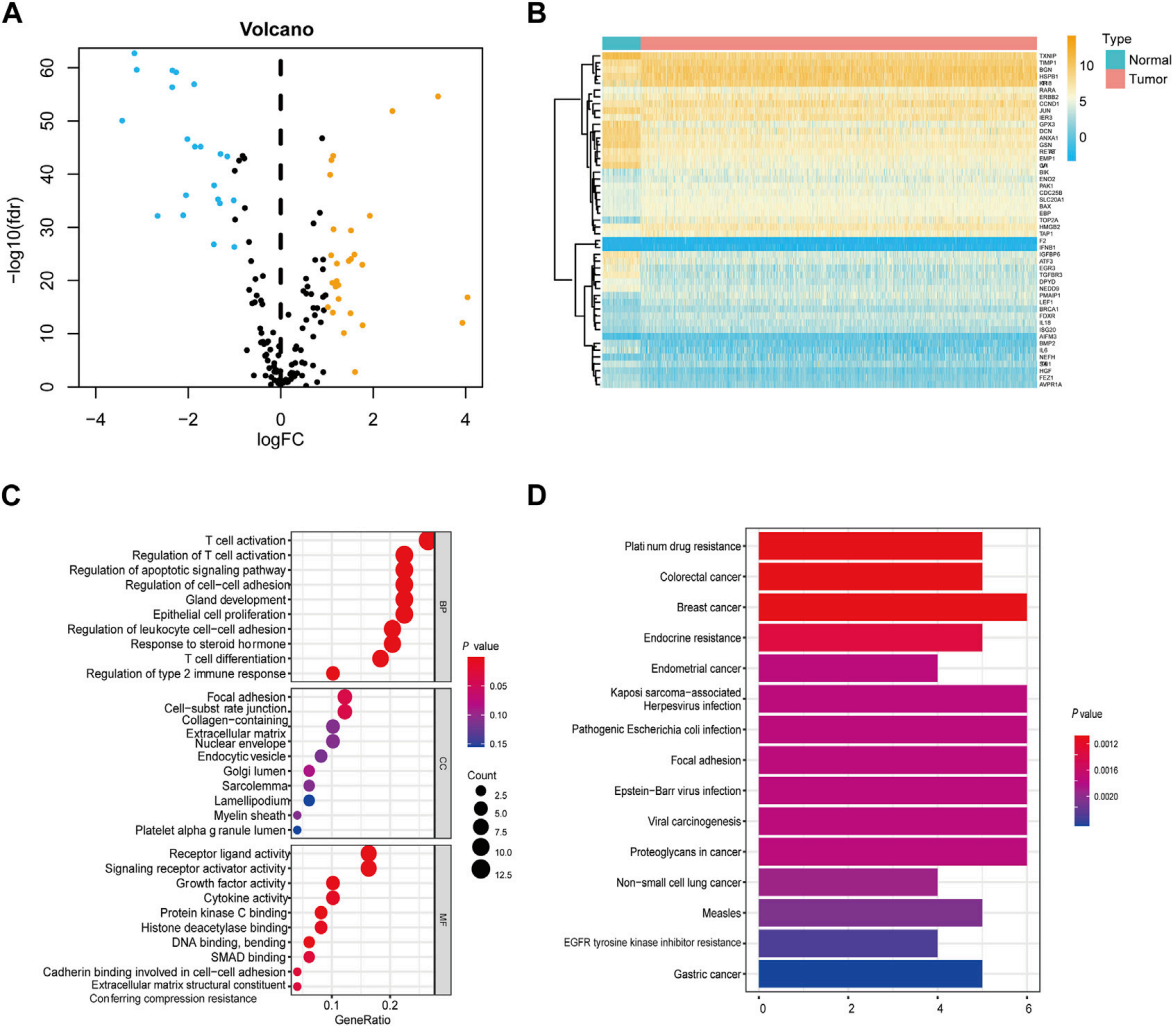
Screening of differentially expressed apoptosis-associated genes in the TCGA cohort **(A)** Volcano plots showing the apoptosis-related DEGs. Yellow represents significantly upregulated genes. Blue indicates significantly downregulated genes. Black shows non-differentially expressed genes. **(B)** Heatmap of differentially expressed apoptosis-related genes relative to normal tissues. **(C)** GO enrichment of apoptosis-related DEGs. **(D)** KEGG pathways of apoptosis-associated DEGs.

Our findings indicate significant differential expression of 49 apoptosis-related genes between tumor samples and their adjacent normal counterparts (Figures 2A, B). Upon scrutinizing the Gene Ontology (GO) category for apoptosis-related genes, we unearthed that the activation of T-cell mediated responses and the regulation of apoptotic pathways were among the most perturbed biological processes. Furthermore, in the Kyoto Encyclopedia of Genes and Genomes (KEGG) analysis, prominent enrichment was observed in cancer-associated pathways (Figures 2C,D; Supplementary Tables S2, S3).

**FIGURE 3:**
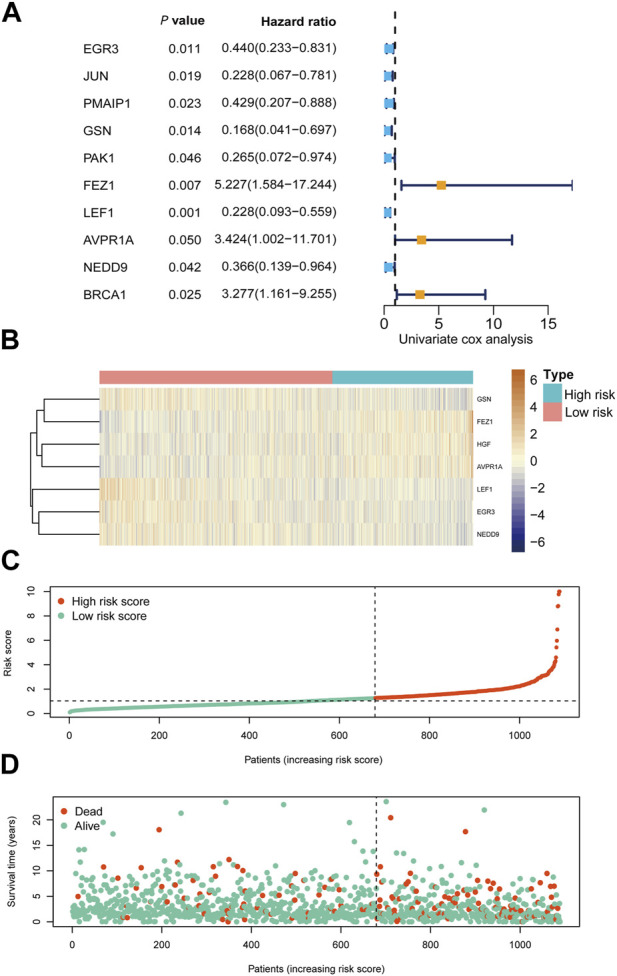
Prognostic analysis of risk score formula in TCGA cohort **(A)** Forest plots showing 10 prognosis-associated genes via univariate cox regression. **(B)** Heat map of the seven-gene signature in the TCGA cohort. **(C)** Distribution of risk score in the TCGA cohort. **(D)** Distribution of survival status of the TCGA cohort patients.

Based on univariate Cox regression analysis, we identified 10 apoptosis-related genes holding prognostic relevance for overall survival (OS) in our patient cohort (Figure 3A; Supplementary Table S4). Among these, FEZ1 appeared to exert the most substantial influence on breast cancer OS, manifesting a hazard ratio (HR) of 1.87 (95% CI: 1.18–2.97, *p* = 0.007). In addition, EGR3, GSN, LEF1, AVPR1A, NEDD9, and HGF emerged as influential contributors to OS, displaying HR values of 0.55 (95% CI: 0.40–0.77, *p* < 0.001), 0.61 (95% CI: 0.44–0.85, *p* = 0.003), 0.60 (95% CI: 0.43–0.82, *p* = 0.001), 2.08 (95% CI: 1.37–3.17, *p* = 0.00046), 0.62 (95% CI: 1.0–2.32, *p* = 0.0045), and 1.60 (95% CI: 1.10–2.32, *p* = 0.0127) respectively, as depicted in Supplementary Figure S1.

### Prognostic evaluation based on RNA-seq profiles

We conducted RNA-seq profiling of the aforementioned 10 genes using stepwise regression analysis. To establish a prognostic model, we identified seven essential genes. Our findings revealed that higher expression levels of EGR3, GSN, LEF1, and NEDD9 were correlated with improved prognosis in breast cancer, while elevated expression of AVPR1A, FEZ1, and HGF yielded contrasting results, as depicted in Supplementary Figure S1. Subsequently, we formulated a prognostic model for breast cancer by incorporating these seven crucial apoptosis-related genes.

The risk score for this model was calculated using the following equation: HGF × 0.650937 - EGR3 × 0.15298 - GSN × 0.46446 + FEZ1 × 0.568178 - LEF1 × 0.3521 + AVPR1A × 0.341397 - NEDD9 × 0.27414.

**FIGURE 4:**
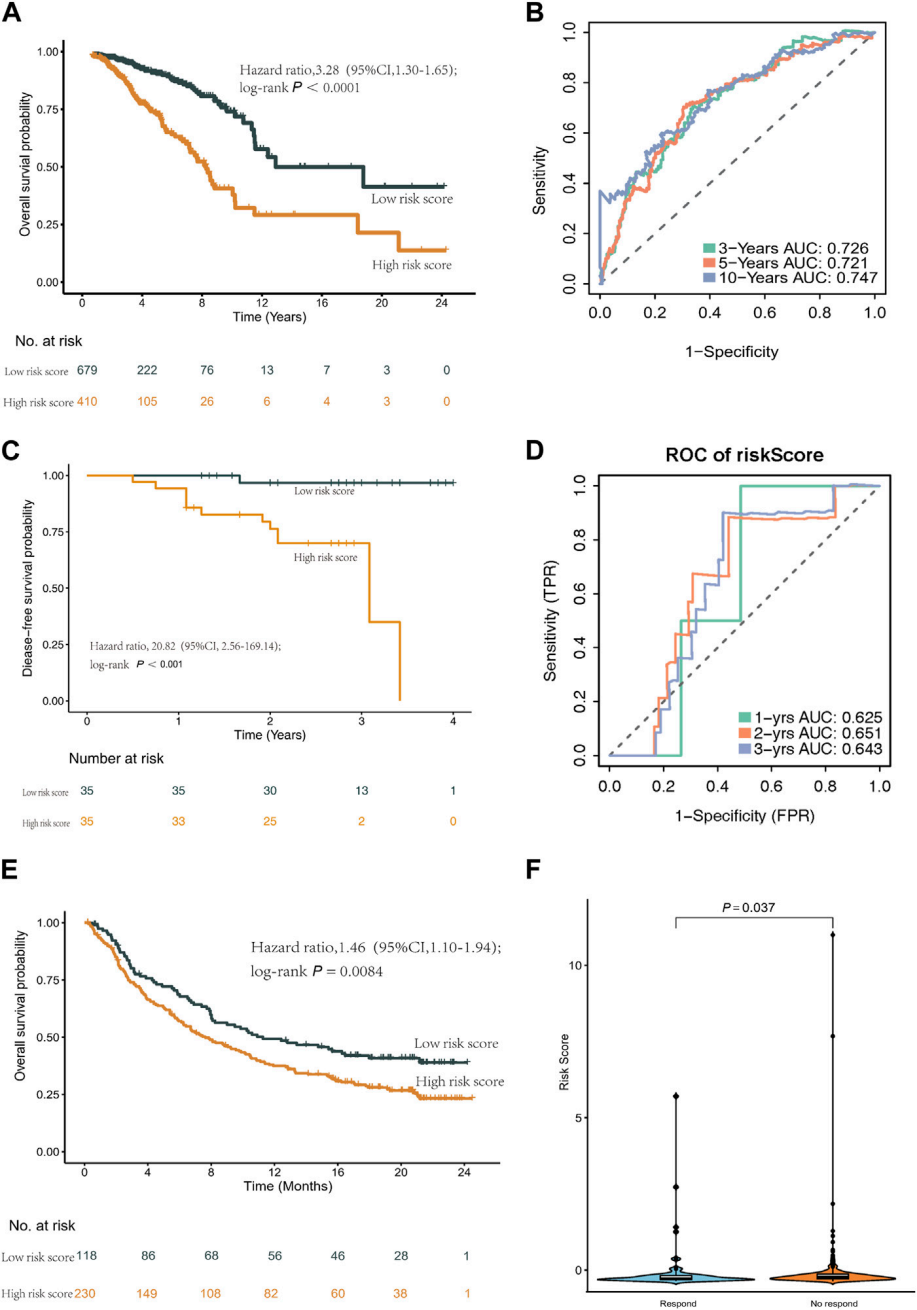
Kaplan-Meier curves and ROC curves of the risk score formula in the TCGA cohort. Validation of the risk score model in SYSMH cohort and immunotherapy cohort IMvigor210. **(A)** Kaplan-Meier curve of patients in the TCGA cohort between the high- and low-risk group patients for OS. **(B)** The ROC analysis proved the prognostic performance of the risk score model in the TCGA cohort. **(C)** Kaplan-Meier curve of patients in the SYSMH cohort between the high- and low-risk group patients for DFS. **(D)** The ROC analysis proved the prognostic performance of the risk score model in the SYSMH cohort. **(E)** Kaplan-Meier survival curve of OS of the high- and low-risk groups in the IMvigor210 cohort. **(F)** The difference in risk score in the subgroup of anti PD-L1 treatment response.

Utilizing this formula, we computed the risk score for each sample and visualized its distribution, as shown in Figure 3B–D. Based on the optimal cut-off point, we categorized patients into a high-risk group (n = 410) and a low-risk group (n = 679). Strikingly, Kaplan-Meier survival curves demonstrated significantly lower overall survival rates in the high-risk group compared to the low-risk group (HR = 3.28, 95% CI = 2.35–4.56, *p* < 0.001) (Figure 4A).

We evaluated the predictive performance of our prognostic model by constructing time-dependent ROC curves, yielding AUCs of 0.726, 0.721, and 0.747 for 3-, 5-, and 10-year time frames, respectively (Figure 4B).

Furthermore, we validated the prognostic model across various subtypes of breast cancer. The Kaplan-Meier curves consistently illustrated a robust association between the prognostic model and overall survival in all subtypes (Supplementary Figure S2). In the Luminal A subtype, the AUCs were 0.733, 0.682, and 0.694 at 3, 5, and 10 years, respectively. For the Luminal B subtype, the AUCs were 0.717, 0.799, and 0.807 at 3, 5, and 10 years, respectively. In the HER2-positive subtype, the AUCs were 0.719, 0.793, and 0.683 at 3, 5, and 10 years, respectively. Lastly, for the TNBC subtype, the predicted AUCs were 0.833, 0.775, and 0.785 at 3, 5, and 10 years, respectively (Supplementary Figure S3).

### Prognostic evaluation of the classifier

To assess the prognostic utility of our classifier, we conducted external validation using the METABRIC cohort and SYSMH cohort. Similarly, patients in these cohorts were stratified into high-risk and low-risk groups based on their gene expression profiles. Consistently, mirroring the results obtained from the TCGA cohort, the Kaplan-Meier survival curve demonstrated significantly poorer prognosis for patients in the high-risk group (HR = 1.46, 95% CI = 1.30–1.65, *p* < 0.001) (Supplementary Figure S4). Furthermore, in the SYSMH cohort, a strong correlation was observed between the risk score and reduced disease-free survival (HR = 20.82, 95% CI = 2.56–169.14, *p* < 0.001) (Figure 4C), with AUC values of 0.625, 0.651, and 0.643 for predicting 1-year, 2-year, and 3-year outcomes, respectively (Figure 4D).

Additionally, a comprehensive analysis was conducted within the IMvigor210 cohort to characterize the prognostic model. Notably, low-risk patients exhibited a higher survival rate (HR = 1.46, 95% CI = 1.10–1.94, *p* = 0.0084) (Figure 4E). Furthermore, high-risk patients in the IMvigor210 cohort displayed increased sensitivity to immunotherapy (Figure 4F).

### Functional enrichment analysis with varying risk scores

**FIGURE 5:**
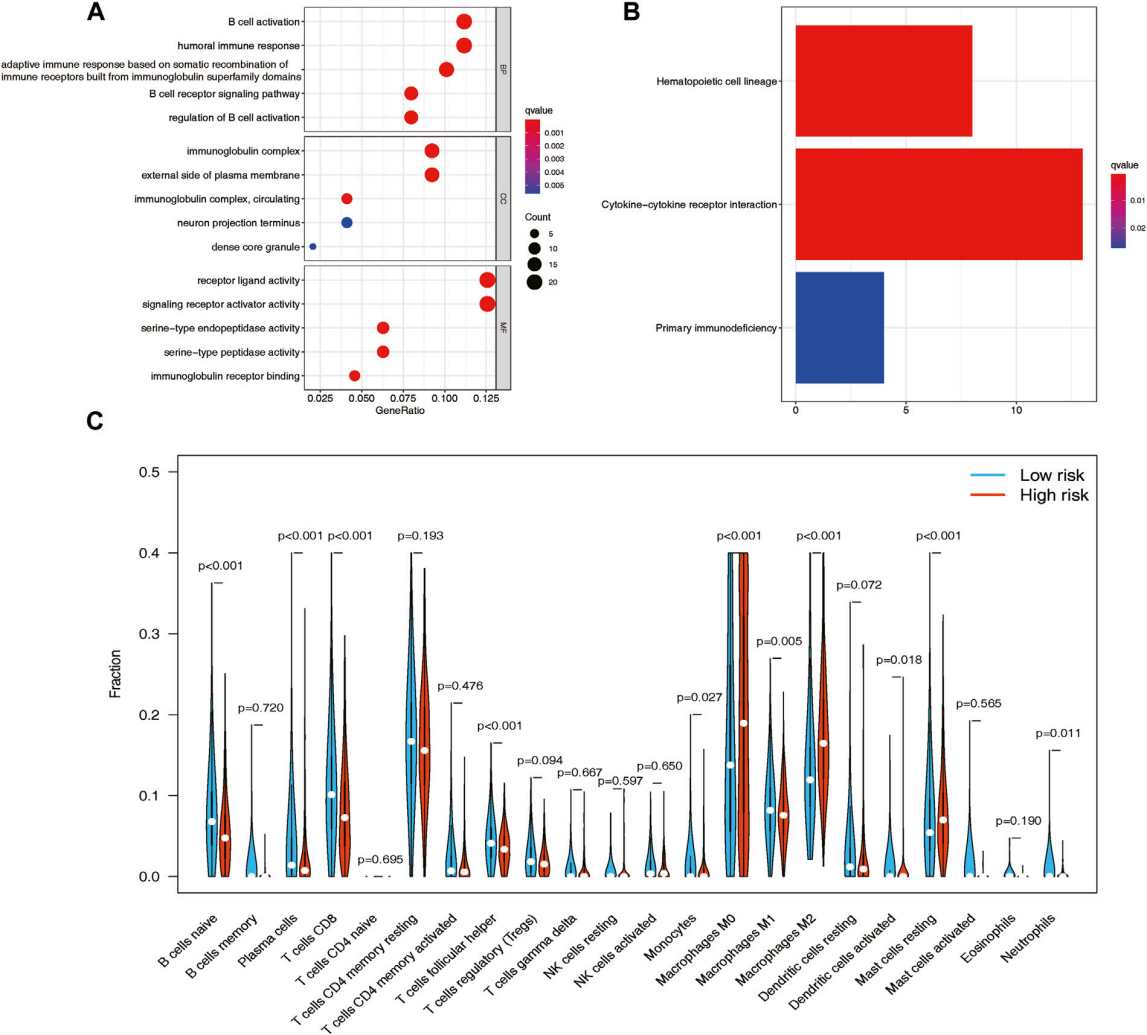
GO and KEGG functional enrichment analyses between the high- and low-risk groups in the TCGA cohort and the analysis of tumor immune infiltrations via CIBERSORT. **(A)** GO enrichment of the DEGs between the high- and low-risk patients in the TCGA cohort. **(B)** KEGG pathways of the DEGs between the high- and low-risk patients in the TCGA cohort. **(C)** Vioplot of 22 immune cell content in the high- and low-risk groups.

Based on the GO functional enrichment analysis, the DEGs between high-risk and low-risk score patients demonstrated significant enrichment in immune-related biological processes (BP) (*p* < 0.005), as illustrated in Figure 5A. Additionally, the KEGG analysis revealed notable differences in cytokine-cytokine receptor interactions between these two patient groups, as depicted in Figure 5B.

### Immune cell infiltration patterns and their impact on overall survival

The CIBERSORT analysis provided an illustration of the proportions of 22 immune cell types, revealing distinct differences in immune cell infiltration patterns among breast cancer samples. To further investigate these distinctions, we conducted the Wilcoxon test to compare immune cell fractions between the high-risk and low-risk groups. The results unveiled significant variations in the proportions of several immune cell types. Specifically, T cells CD8 (*p* < 0.001), resting mast cells (*p* < 0.001), plasma cells (*p* < 0.001), macrophages M0 (*p* < 0.001), naive B cells (*p* < 0.001), and macrophages M2 (*p* < 0.001) exhibited markedly different distributions between the high-risk and low-risk groups. Interestingly, the low-risk group displayed higher levels of infiltration of B cells, plasma cells, and CD8 T cells, while the high-risk group demonstrated elevated levels of infiltration of macrophages M0, macrophages M2, and resting mast cells (Figure 5C). Furthermore, patients with higher levels of naive B cells (*p* < 0.0001) and plasma cells (*p* < 0.0001), as well as lower levels of macrophages M0 (*p* = 0.013) and macrophages M2 (*p* < 0.0001), exhibited a strong association with improved overall survival, as demonstrated by Kaplan-Meier curve analysis (Supplementary Figure S5).

### Evaluation of tumor-infiltrating immune cells and immune function

**FIGURE 6:**
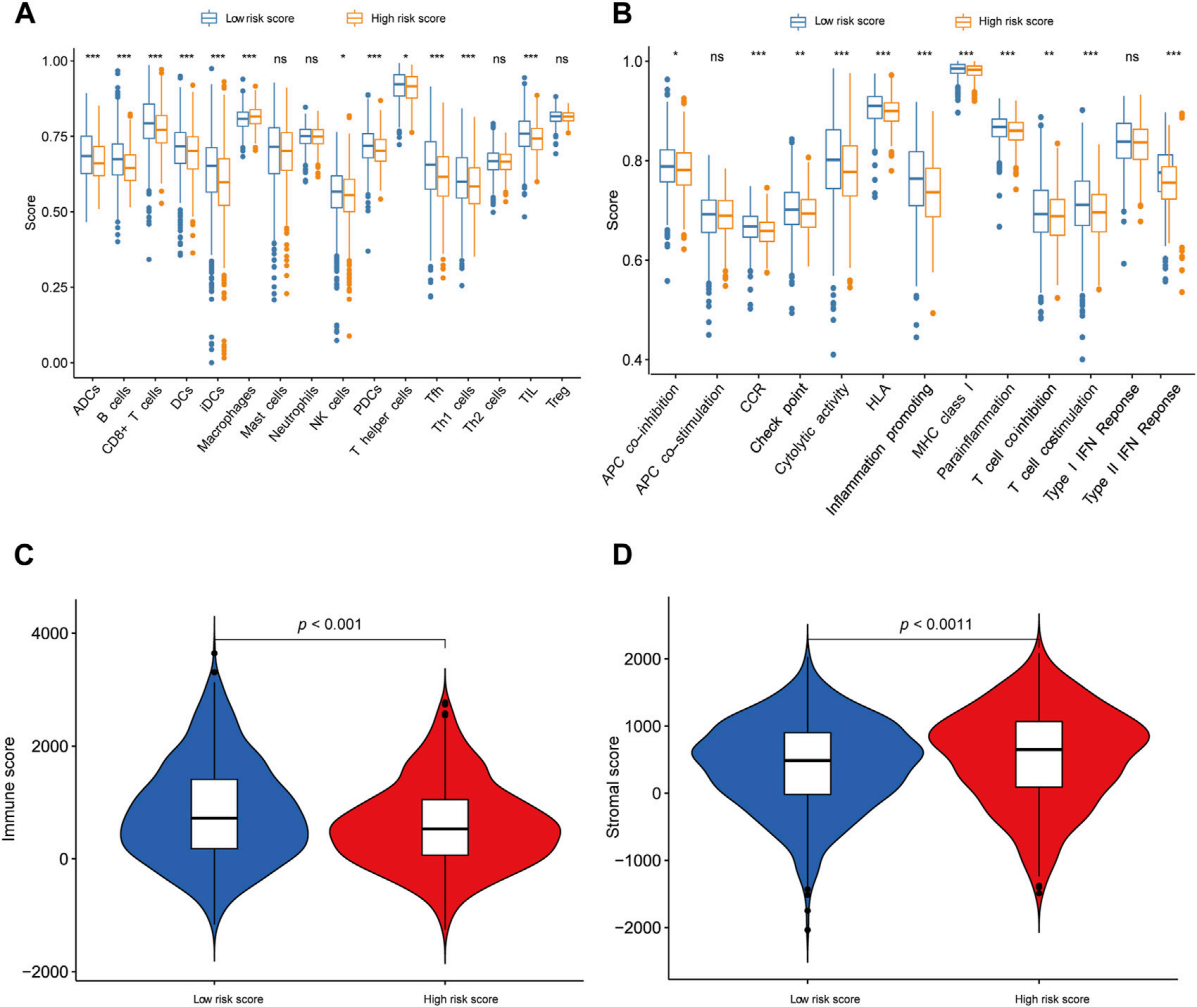
Exploration of tumor immune microenvironment **(A, B)** ssGSEA for the association between immune cell subpopulations and immune-related functions in the high- and low-risk groups. **(C)** The difference of immune score in the high- and low-risk groups. **(D)** The difference in stromal score in the high- and low-risk groups. *p < 0.05; **p < 0.01; ***p < 0.001; ns, not significant.

Through the application of single-sample Gene Set Enrichment Analysis (ssGSEA), our objective was to evaluate the interplay between tumor-infiltrating immune cells and their immune functionality. The findings corroborated the observations made by CIBERSORT, revealing heightened B cell expression in low-risk patients and reduced macrophage expression in the low-risk group. Additionally, ssGSEA analysis unveiled further disparities between the low-risk and high-risk groups, encompassing cytolytic activity, chemokine receptor expression (CCR), MHC class I, HLA (human leukocyte antigen) genes, parainflammation, T cell co-stimulation, type II interferon (IFN) responses, and pro-inflammatory factors (refer to Figures 6A,B). Notably, the low-risk group exhibited a noteworthy increase in immune scores, while the high-risk group displayed a significant elevation in matrix scores (Figures 6C,D).

### Mutation analysis and risk stratification by tumor location

**FIGURE 7:**
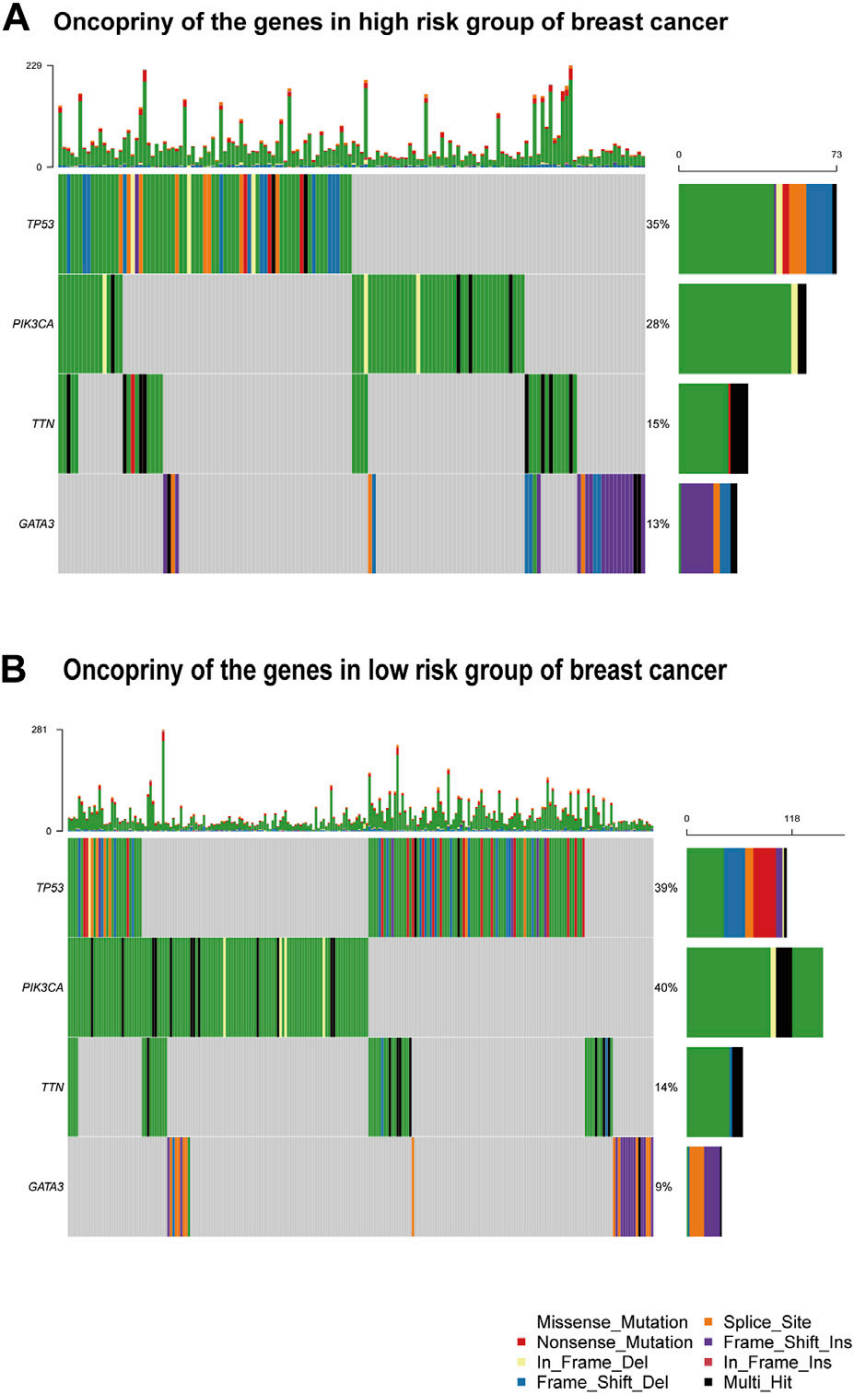
Gene mutation analysis **(A)** Oncoprint of the genes in the high-risk group. **(B)** Oncoprint of the genes in the low-risk group.

Figures 7A, B provide visual representations of the mutation profiles and the distribution of high-risk and low-risk groups based on tumor location for differentially mutated genes. The mutations were subjected to further categorization based on various criteria, with missense mutations being the predominant type. Notably, a noteworthy elevation in the mutation rate of PIK3CA was observed in the low-risk group, underscoring its potential as a target for precision therapeutic interventions (Willis et al., 2020). Additionally, distinctive mutation patterns were discerned among genes associated with triple-negative breast cancer in both the high-risk and low-risk groups (Supplementary Figure S6A, B).

### Prognostic nomogram for predicting breast cancer outcome

**FIGURE 8:**
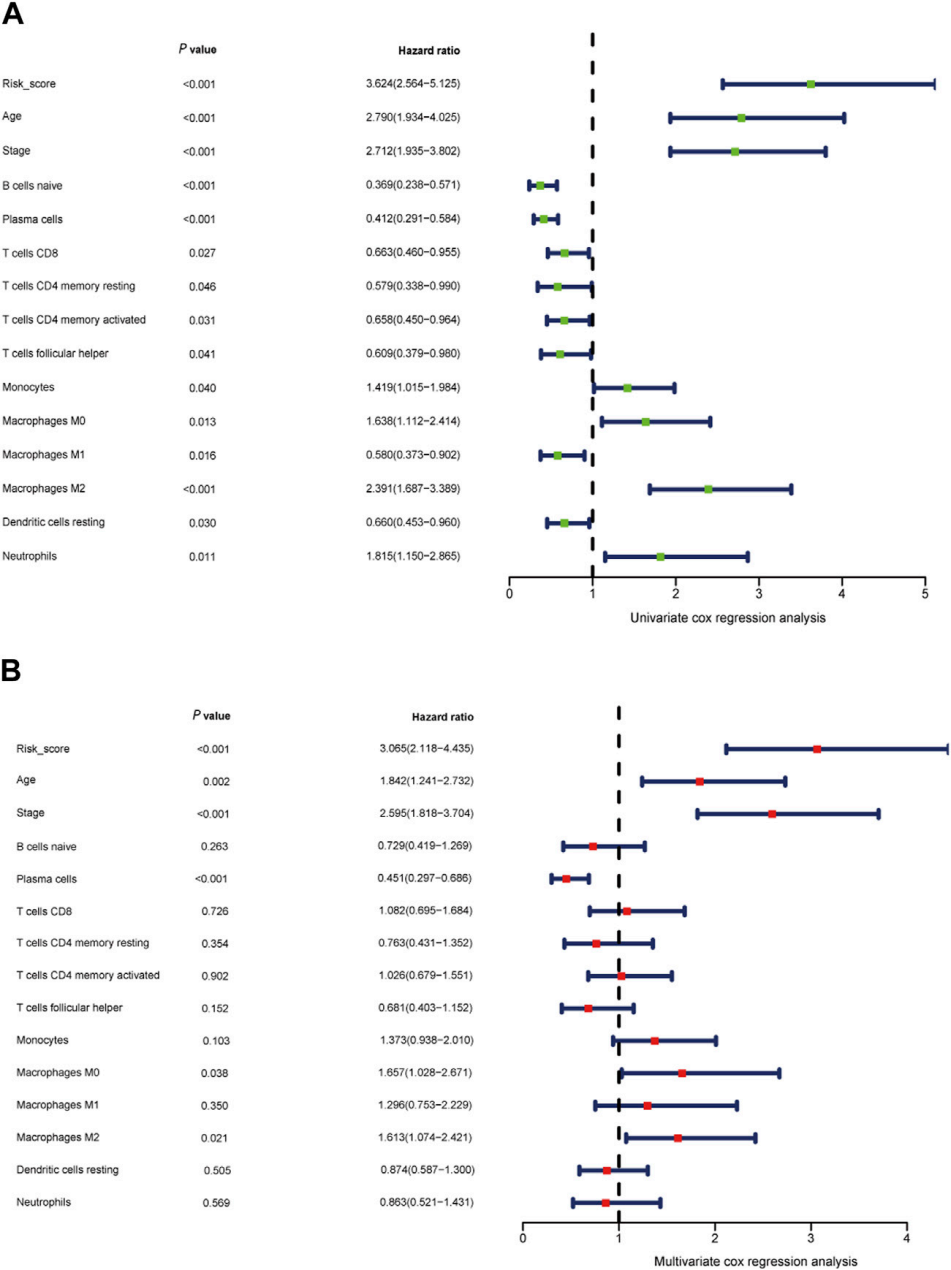
Independent prognostic role of the gene signature **(A, B)** Univariate Cox regression analysis and multivariate Cox regression analysis of risk score model, clinical indicators, and immune cells.

After accounting for other conventional clinical variables within the TCGA dataset, we conducted univariate regression analysis to assess whether the prognostic model could maintain its status as an independent predictor. This analysis aimed to evaluate the predictive utility of clinical characteristics for breast cancer patients. Our findings revealed that the following factors significantly impacted overall survival (OS): age, tumor stage, B cell naive, plasma cells, CD8 T cells, CD4 T cell memory resting, monocytes, macrophages M0, macrophages M1, macrophages M2, CD4 T cell memory activated, dendritic cell resting, neutrophils, and the risk score model (Figure 8A).

To establish a quantitative prognostic assessment method with clinical relevance in breast cancer, we subsequently conducted multivariate regression analysis. This analysis demonstrated that age (*p* = 0.002), tumor stage (*p* < 0.001), plasma cells (*p* < 0.001), macrophages M0 (*p* = 0.038), macrophages M2 (*p* = 0.021), and the risk score (*p* < 0.001) all emerged as independent prognostic factors strongly associated with OS (Figure 8B).

**FIGURE 9:**
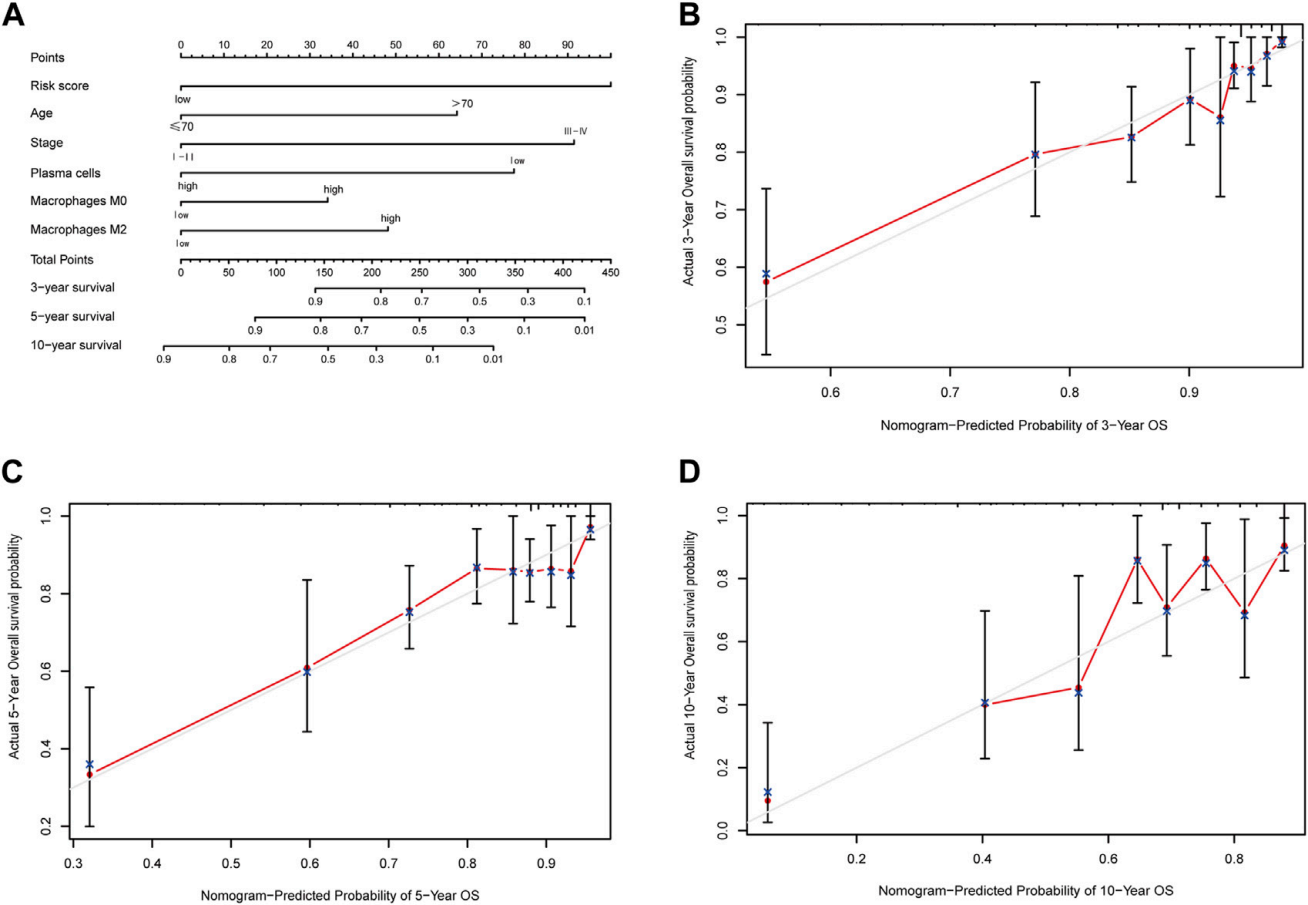
Nomogram predicting overall survival of breast cancer patients and the calibration curve for the nomogram. **(A)** Nomogram predicting overall survival of breast cancer patients. **(B)** Calibration curve of the nomogram for 3-year prediction. **(C)** Calibration curve of the nomogram for 5-year prediction. **(D)** Calibration curve of the nomogram for 10-year prediction.

Building upon the prognostic model centered around apoptosis-related genes, conventional clinical features, and multi-omics immune cell data, we crafted a quantitative prognostic evaluation nomogram for breast cancer (Figure 9A). This nomogram provided a vertical axis scale for estimating 3-year, 5-year, and 10-year OS probabilities, with the predicted outcomes closely aligning with actual results, thus validating the nomogram’s accuracy (Figures 9B–D).

**FIGURE 10:**
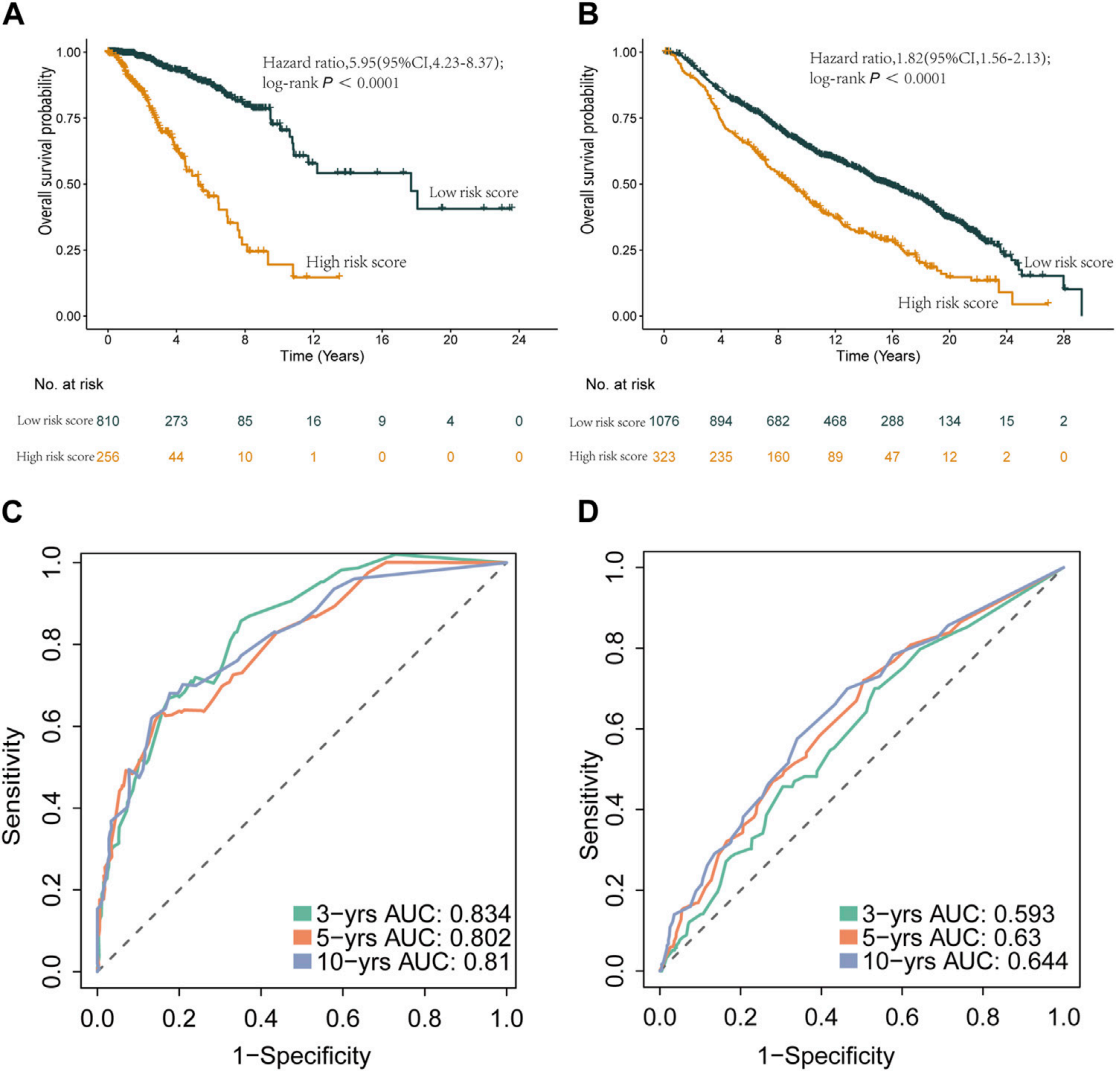
Kaplan-Meier curves and ROC curves of the two groups reclassified by nomogram score. **(A)** Kaplan-Meier curve of patients in the TCGA cohort between the high- and low-risk group patients for OS. **(B)** Kaplan-Meier curve of patients in METABRIC cohort between the high- and low-risk group patients for OS. **(C)** The ROC analysis proved the prognostic performance of the risk score model in the TCGA cohort. **(D)** The ROC analysis proved the prognostic performance of the risk score model in in METABRIC cohort.

Furthermore, we applied the nomogram to compute scores for each patient in both the TCGA and METABRIC cohorts, classifying them into two groups using an optimal cutoff value. Survival analysis showcased significant distinctions between these two groups, surpassing the results derived solely from the risk score model (Figure 10). Importantly, the prognostic nomogram demonstrated superior performance over the use of a single prognostic variable, exhibiting high accuracy and long-term predictive capability extending up to 10 years (Supplementary Figure S7).

Additionally, we assessed the DEGs between the two patient groups based on the nomogram scores. Our analysis highlighted that the most significant alterations within the biological process GO category were related to the humoral immune response. Moreover, KEGG pathway analysis unveiled significant enrichment in pathways associated with hematopoietic cell lineage and protein digestion and absorption (Supplementary Figure S8; Supplementary Table S6). In summary, our study underscores that the prognostic nomogram offers a more precise and dependable method for forecasting the prognosis of breast cancer patients.

### qPCR analysis to evaluated mRNA expression level

To identify differential gene expression within the prognostic model, we conducted a gene expression analysis across various breast cancer cell lines and a normal mammary epithelial cell line, MCF-10A. The selected cell lines encompassed the luminal A cell line, MCF-7, the luminal B cell line, BT-474, the Her2-overexpressed cell line, SK-BR-3, and the triple-negative breast cancer cell line, MDA-MB-231. Quantitative PCR (qPCR) was employed, and the relevant primer information is summarized in Supplementary Table S7.

**FIGURE 11:**
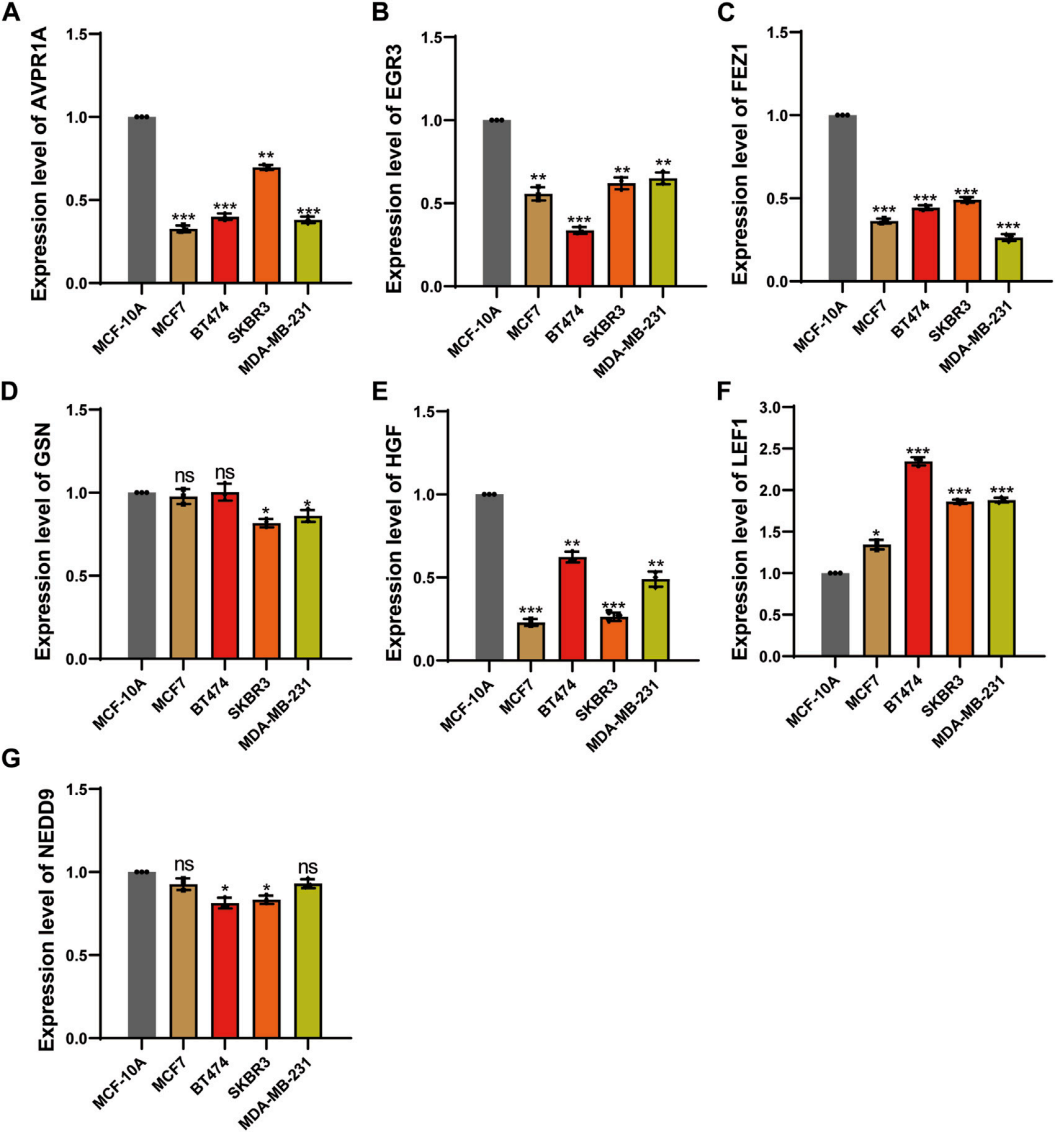
qPCR analysis to evaluated mRNA expression level. **(A)** The expression level of AVPR1A. **(B)** The expression level of EGR3. **(C)** The expression level of FEZ1. **(D)** The expression level of GSN. **(E)** The expression level of HGF. **(F)** The expression level of LEF1. **(G)** The expression level of NEDD9. *p < 0.05; **p < 0.01; ***p < 0.001; ns, not significant.

The results revealed a notable downregulation of AVPR1A, FEZ1, HGF, GSN, NEDD9, and EGR3 expression in the breast cancer cell lines (MCF7, BT-474, SK-BR-3, MDA-MB-231) when compared to the normal mammary epithelial cell line (MCF-10A). In contrast, LEF1 expression exhibited an increase relative to MCF-10A (Figure 11).

## Discussion

In this study, we conducted a comprehensive analysis of genomic data derived from breast cancer tissues and normal mammary tissues to investigate the differential expression of apoptosis-related genes, with the aim of identifying potential biomarkers for breast cancer. Subsequently, we developed and rigorously validated a prognostic model for breast cancer, leveraging the expression profiles of seven apoptosis-related genes. Furthermore, we stratified breast cancer patients into low- and high-risk categories based on their respective risk scores. Our results unveiled substantial disparities in prognosis, molecular pathways, the tumor microenvironment (TME), immune functionality, and gene mutational profiles between these low-risk and high-risk cohorts. Significantly, the low-risk group demonstrated a more favorable response to immunotherapy. To synthesize the risk score model, clinical variables, and immune cell data, we formulated a multi-omics nomogram. Calibration plots and area under the curve (AUC) analyses affirmed the improved predictive efficacy of this nomogram.

Apoptosis constitutes a pivotal mechanism that regulates cell proliferation by controlling mutation rates, thereby curbing malignant transformations. The inhibition of specific apoptosis signaling pathways can lead to treatment resistance. Extensive research has highlighted the association between apoptosis mechanisms and the efficacy of immunotherapy, indicating that antagonists of apoptosis antagonistic proteins can enhance the effectiveness of cancer immunotherapy (Michie et al., 2020). In our previous investigations, we established a novel immune classification based on long non-coding RNAs (lncRNAs) and cytotoxic T lymphocyte (CTL) infiltration. This classification delineated four distinct immune subtypes in the context of clinical cancer immunotherapy. Moreover, multi-omics panels incorporating CTL infiltration, tumor mutation burden, PD-L1 expression, and lncRNA profiles have proven to be practical biomarkers for cancer immunotherapy (Yu et al., 2020b). Consequently, our study employed mRNA profiling to establish a prognosis model founded on seven apoptotic genes, which we subsequently validated in cancer patients undergoing immunotherapy. Notably, patients classified in the low-risk group demonstrated a more favorable therapeutic response to anti-PD-L1 therapies compared to their high-risk counterparts.

Our previous research identified a correlation between the response to immune checkpoint inhibitor therapy and the status of MUC16 variants (Yu et al., 2020c). Furthermore, inhibiting MUC16 has been shown to suppress apoptosis in triple-negative breast cancer (TNBC) cells (Lakshmanan et al., 2012). In the present study, we harnessed seven apoptotic genes to construct a prognostic model capable of effectively predicting the overall survival (OS) of breast cancer patients. We subjected this model to internal validation across various breast cancer subtypes and external validation using the METABRIC cohort. Additionally, our research entailed univariate Cox analysis, stepwise regression analysis, and enrichment analysis, among other analytical methods, to address the limitations of previous clinical prediction models, offering valuable insights for the clinical management of breast cancer patients.

The tumor microenvironment (TME), which plays a pivotal role in immune surveillance, can serve as a prognostic factor in breast cancer (He et al., 2020). Immune biomarkers have been established as crucial prognostic factors for overall survival in cancer patients (Yu et al., 2019). Research indicates that immune cells within the tumor microenvironment can both promote and inhibit cancer cell apoptosis. For instance, cytotoxic T lymphocytes and natural killer cells induce apoptosis in tumor cells through the secretion of granzymes and the expression of death ligands like FasL and TRAIL (Dotiwala et al., 2016). Conversely, certain tumor-associated macrophages and regulatory T cells can suppress apoptosis by releasing factors that enhance cancer cell survival and resistance to cell death (Chen et al., 2024). This complex interplay influences cancer progression, therapeutic response, and the efficacy of treatments targeting immune checkpoints or apoptosis pathways, underscoring the importance of understanding the mechanisms governing these interactions for the development of effective cancer therapies (Hänggi and Ruffell, 2023). Macrophages, when in a resting (M0) state, can polarize into either M1 or M2 macrophages upon stimulation by specific tumor factors. Extensive research has focused on M2 macrophages due to their immunosuppressive nature and their contribution to tumor growth and angiogenesis (Chen et al., 2017). Elevated plasma cell levels have been associated with a more favorable prognosis in breast cancer, whereas higher M0 and M2 macrophage levels are linked to a poorer prognosis (Chávez-Galán et al., 2015; Dai et al., 2021). Our study delved into the characteristics of apoptotic genes and the tumor microenvironment in breast cancer, providing a robust framework for prognostic prediction and immunotherapy sensitivity assessment. Additionally, we found that high-risk patients based on the apoptotic gene model exhibited unfavorable immune cell infiltration, such as macrophages M0 and M2. Verification through the IMvigor210 immunotherapy cohort indicated poorer responsiveness to immunotherapy among high-risk patients, offering comprehensive insights into the interplay of immune cell dynamics, tumor apoptosis, and treatment outcomes in breast cancer.

To enhance the precision of OS prediction in breast cancer, we integrated the risk score with two clinical variables (age and stage) and immune cell populations (plasma cells, M0, and M2 macrophages) to construct a multi-omics nomogram. ROC analysis demonstrated significant improvements in the nomogram’s predictive performance upon the inclusion of the risk score in conjunction with clinical characteristics and immune cell data. Furthermore, the calibration plot showcased robust agreement between the predicted outcomes and observed results. Of particular note, our prognostic model exhibited exceptionally reliable predictions for triple-negative breast cancer (TNBC), a subtype characterized by high mortality and resistance to conventional therapies (Yin et al., 2020). In recent years, immunotherapy has emerged as an effective treatment approach for TNBC (Keenan and Tolaney, 2020). Clinical trials like KEYNOTE-012 (Nanda et al., 2016), evaluating pembrolizumab treatment in TNBC patients, have shown promising overall response rates, further underscoring the potential utility of the risk score model as a predictive marker for TNBC patients undergoing immunotherapy. Our previous retrospective study, which encompassed 1,088 breast cancer patients, identified early invasive breast cancer through a comprehensive assessment combining axillary lymph node and tumor area clinicopathological characteristics with molecular subtypes and MRI multi-sequence key radiological features (Yu et al., 2021). However, this prior study had limitations, particularly the absence of genetic characterization in breast cancer. In the current study, we delved into the characteristics of apoptotic genes and the tumor microenvironment in breast cancer, thereby providing a robust framework for prognostic prediction and immunotherapy sensitivity assessment in breast cancer patients.

It is important to acknowledge several limitations within our study. Firstly, our study is based on retrospective samples, warranting further validation with prospective data. Additionally, the absence of immunotherapy-treated patients in the TCGA cohort limits our ability to fully assess the applicability of apoptosis biomarkers. Future investigations should consider the incorporation of additional features, such as neoantigen load, long non-coding RNA expression, and microRNA profiles, to further enhance the accuracy and interpretability of multi-biomarker sets.

## Conclusion

In this study, the prognostic model, using seven apoptosis-related genes, accurately predicts survival for various breast cancer types and suggests these genes as targets for treatments. It identified the breast cancer patients into high or low-risk groups, showing significant differences in pathways, immune microenvironment between these two groups. Notably, there’s a association between our prognostic score and response to anti-PD-L1thearpy, highlighting its value in cancer treatment. By combining risk scores with clinical data and immune profiles, we’ve developed a multi-factor nomogram, greatly improving our predictions’ precision.

## Data Availability

The original contributions presented in the study are publicly available. This data can be found here in the GEO database https://www.ncbi.nlm.nih.gov/geo/, accession number GSE189371.
