# Supplementary material for: Deciphering the role of apoptosis signature on the immune dynamics and therapeutic prognosis in breast cancer: Implication for immunotherapy
Source: Front Genet. 2024 May 2;15:1332935. doi: 10.3389/fgene.2024.1332935 (PMC11097162; doi:10.3389/fgene.2024.1332935)
Supplement: Supplementary file 1 [file Table1.DOCX]

**Supplementary figures**

**Fig. S1.** Kaplan-Meier curve of seven apoptosis-related differential genes in breast cancer

**Fig. S2.** Kaplan-Meier curves of the risk score model in different subtypes of breast cancer

**Fig. S3.** Time-related receiver operating characteristic curve analysis in different subtypes of breast cancer

**Fig. S4.** Validation of the risk score signature in the METABRIC cohort

**Fig. S5.** Kaplan-Meier survival curve in various types of immune cells.

**Fig. S6.** Gene mutation analysis of triple-negative breast cancer**.**

**Fig. S7.** Time-related ROC curve for the nomogram and each factor.

**Fig. S8.** GO and KEGG functional enrichment analyses between two groups according to the nomogram score in TCGA.

**Fig. S1.** Kaplan-Meier survival curve of seven apoptosis-related differential genes in breast cancer.


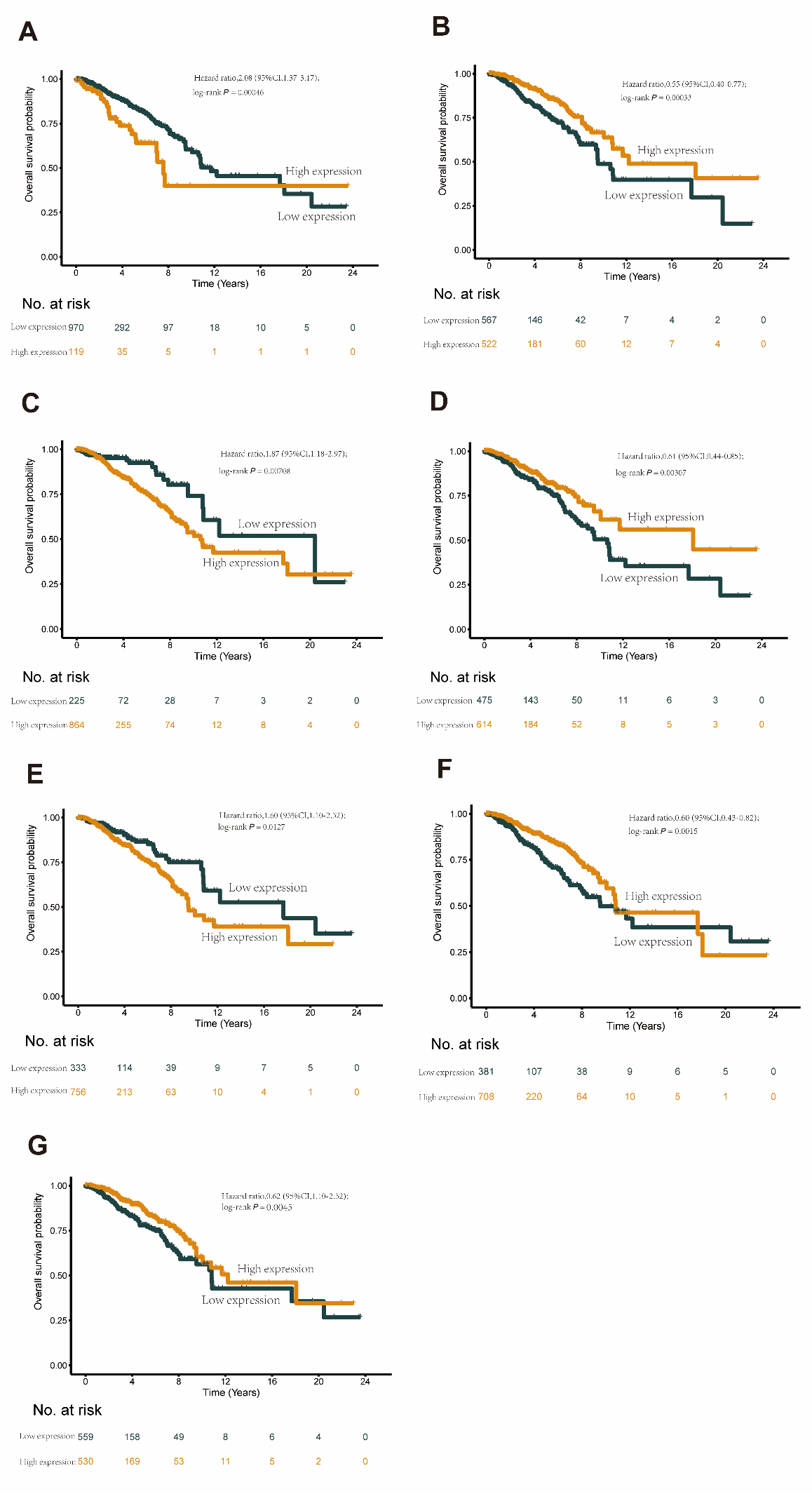


(A) Kaplan-Meier survival curve of AVPR1A expression. (B) Kaplan-Meier survival curve of EGR3 expression. (C) Kaplan-Meier survival curve of FEZ1 expression. (D) Kaplan-Meier survival curve of GSN expression. (E) Kaplan-Meier survival curve of HGF expression. (F) Kaplan-Meier survival curve of LEF1 expression. (G) Kaplan-Meier survival curve of NEDD9 expression.

**Fig. S2.** Kaplan-Meier survival curves of the risk score model in different subtypes of breast cancer.

**
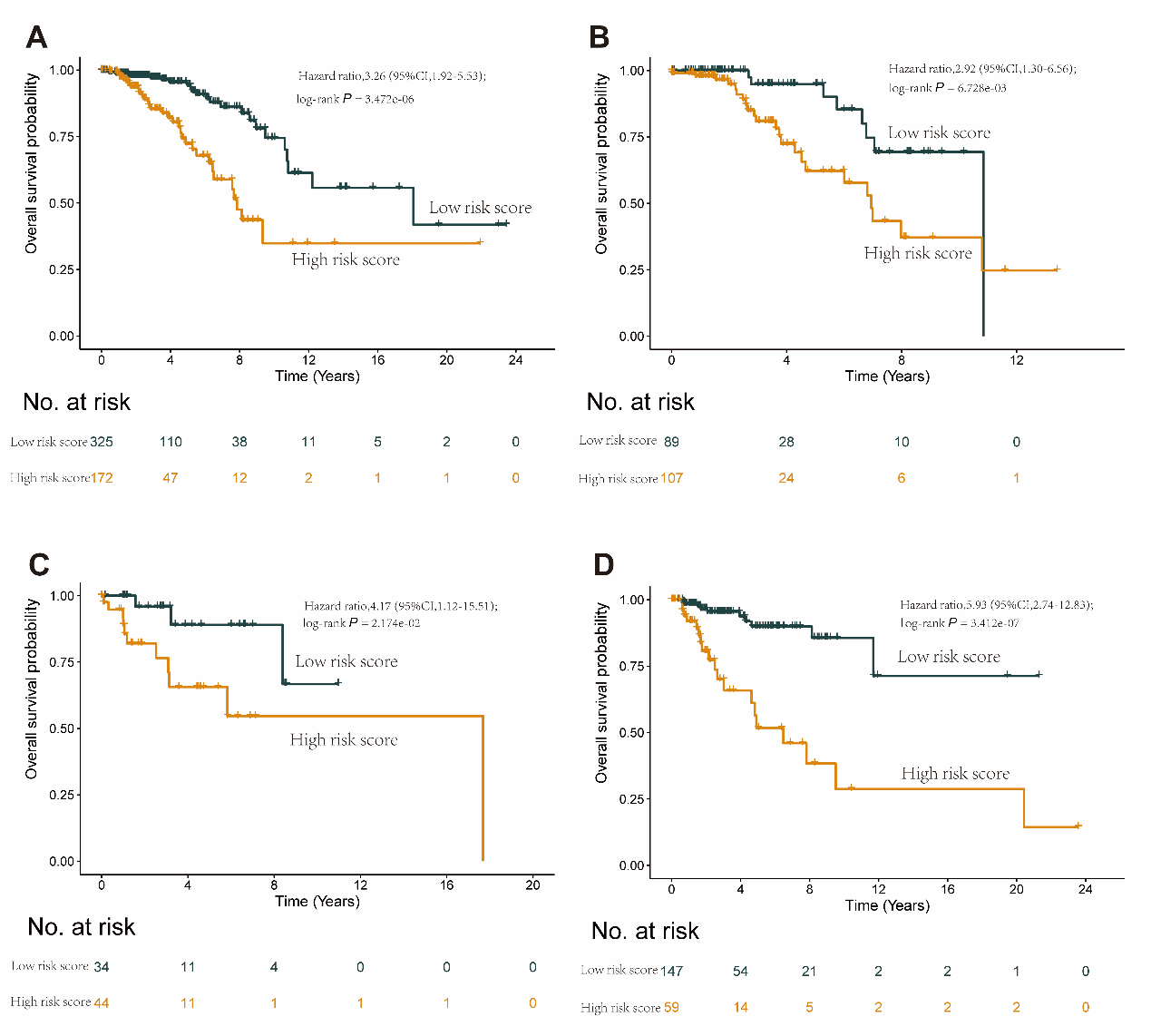
**

(A) Kaplan-Meier survival curve of the patients between the high-risk and low-risk groups for OS in Luminal A subtype.(B) Kaplan-Meier survival curve of the patients between the high-risk and low-risk groups for OS in Luminal B subtype.(C) Kaplan-Meier survival curve of the patients between the high-risk and low-risk groups for OS in HER2-positive subtype.(D) Kaplan-Meier survival curve of the patients between the high-risk and low-risk groups for OS in triple-negative breast cancer.

**Fig. S3.** Time-related receiver operating characteristic curve analysis in different subtypes of breast cancer.

**
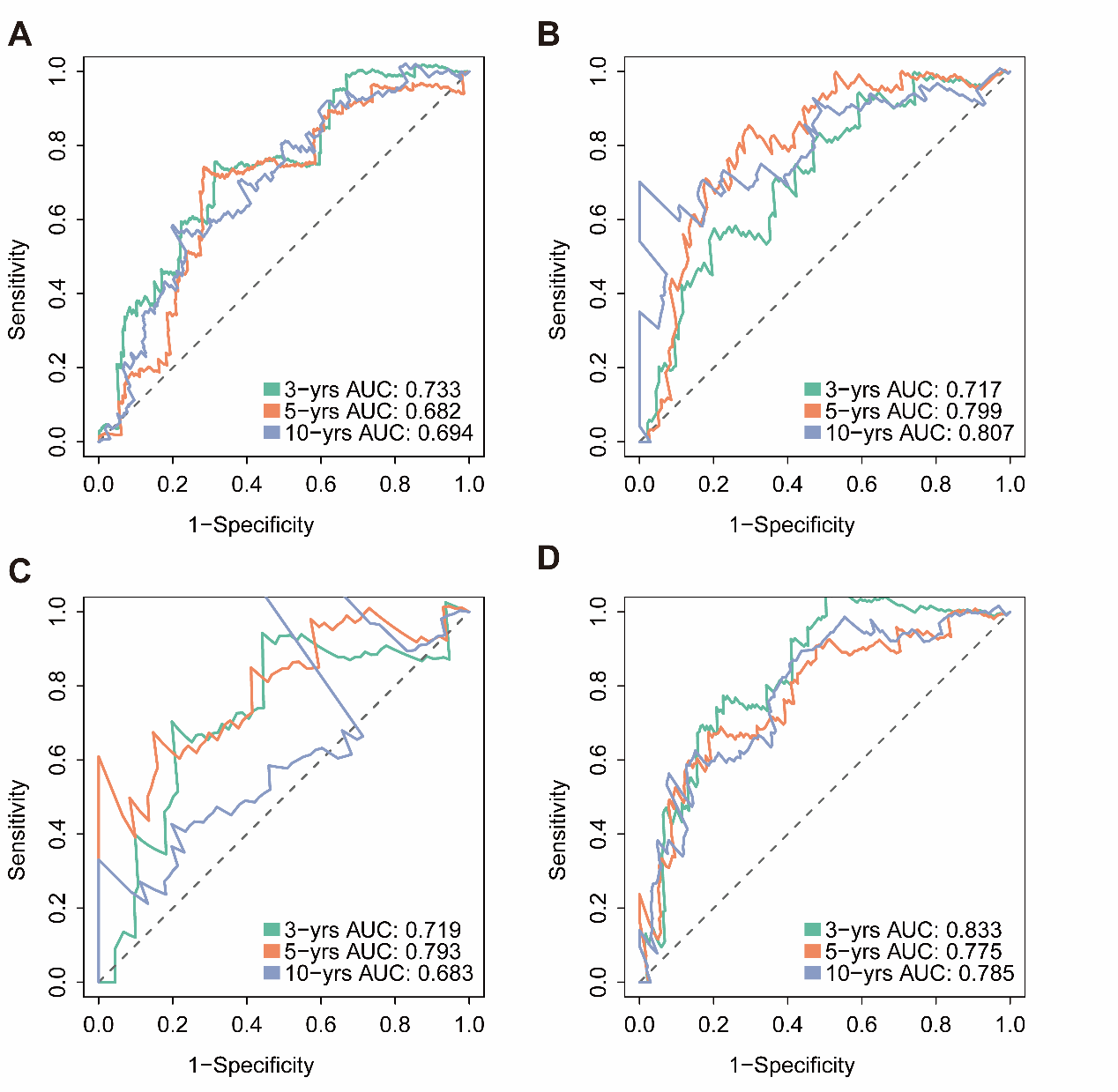
**

(A) Time-related ROC analysis of Luminal A subtype. (B) Time-related ROC analysis of Luminal B subtype. (C) Time-related ROC analysis of HER2-positive subtype. (D) Time-related ROC analysis of triple-negative breast cancer.

**Fig.S4.** Validation of the risk score signature in the METABRIC cohort

**
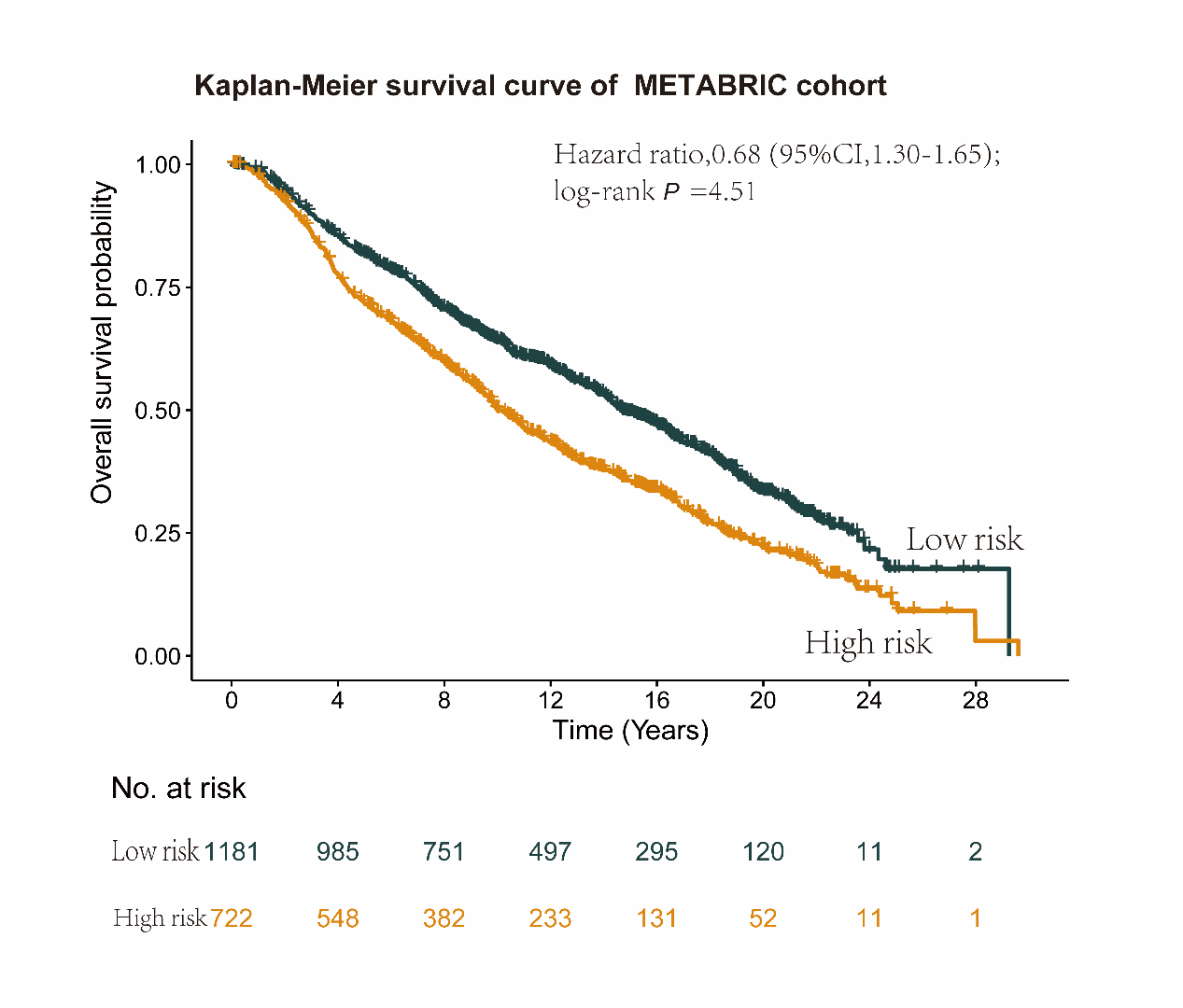
**

Kaplan-Meier survival curve of the patients between the high-risk and low-risk groups for OS in METABRIC cohort.

**Fig. S5.** Kaplan-Meier survival curve in various types of immune cells.

**
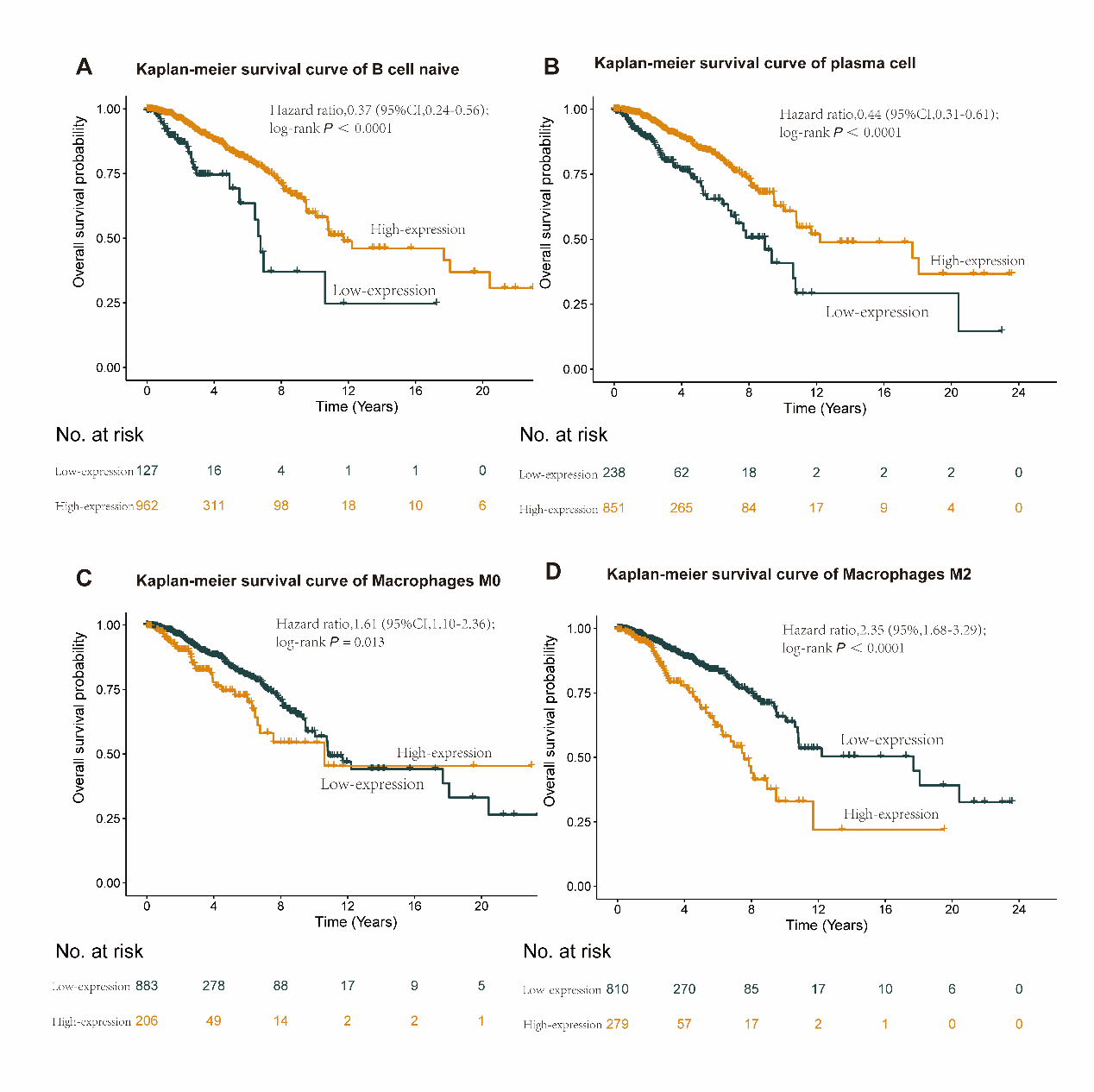
**

(A) Kaplan-Meier survival curve of B cell naive expression. (B) Kaplan-Meier survival curve of plasma cell expression. (C) Kaplan-Meier survival curve of Macrophages M0 expression. (D) Kaplan-Meier survival curve of Macrophages expression.

**Fig. S6.** Gene mutation analysis of triple-negative breast cancer**.**

**
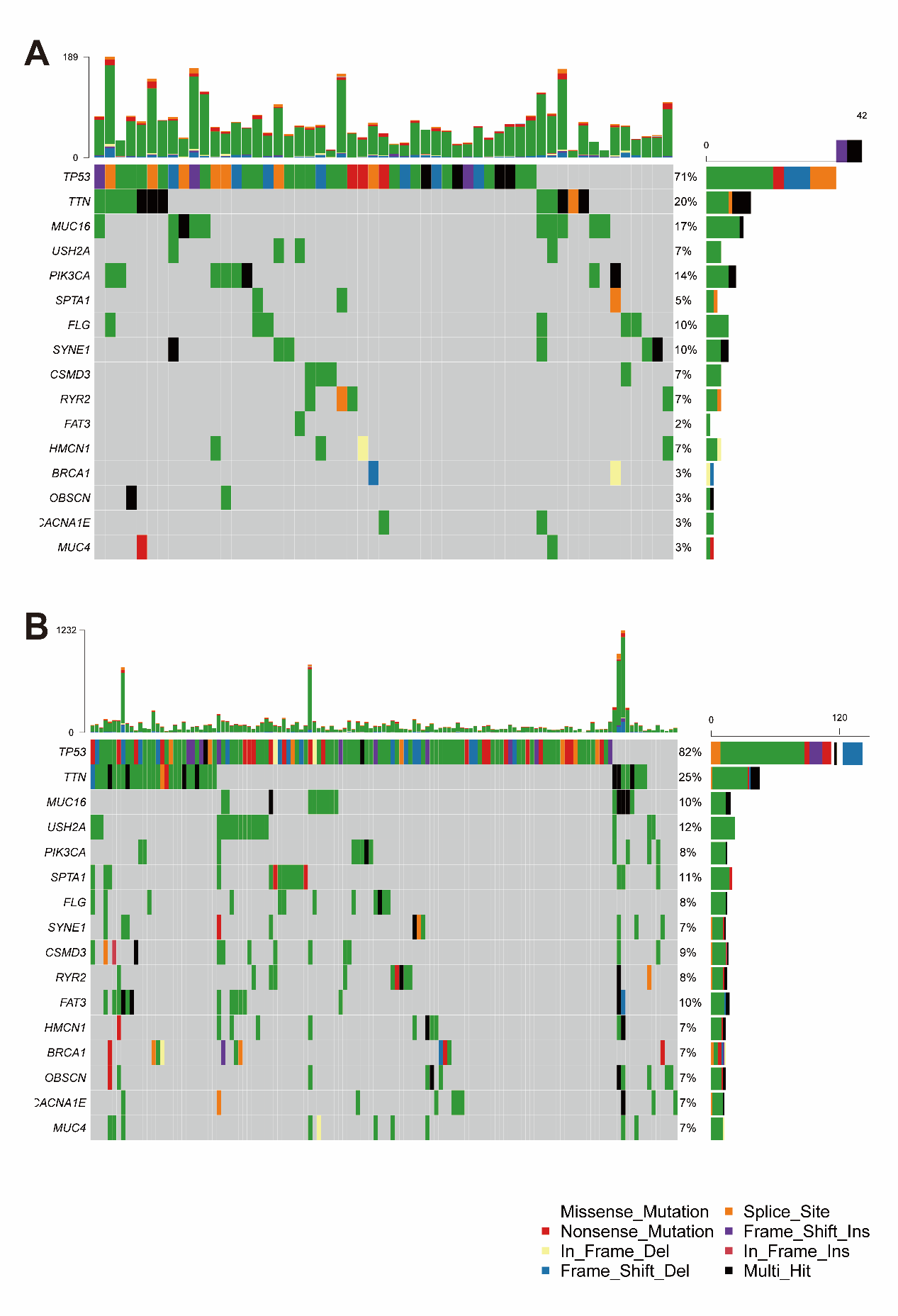
**

(A) Oncoprint of the top 30 genes in high-risk group in triple-negative breast cancer. (B) Oncoprint of the top 30 genes in low-risk group in triple-negative breast cancer.

**Fig. S7.** Time-related ROC curve for the nomogram and each factor.


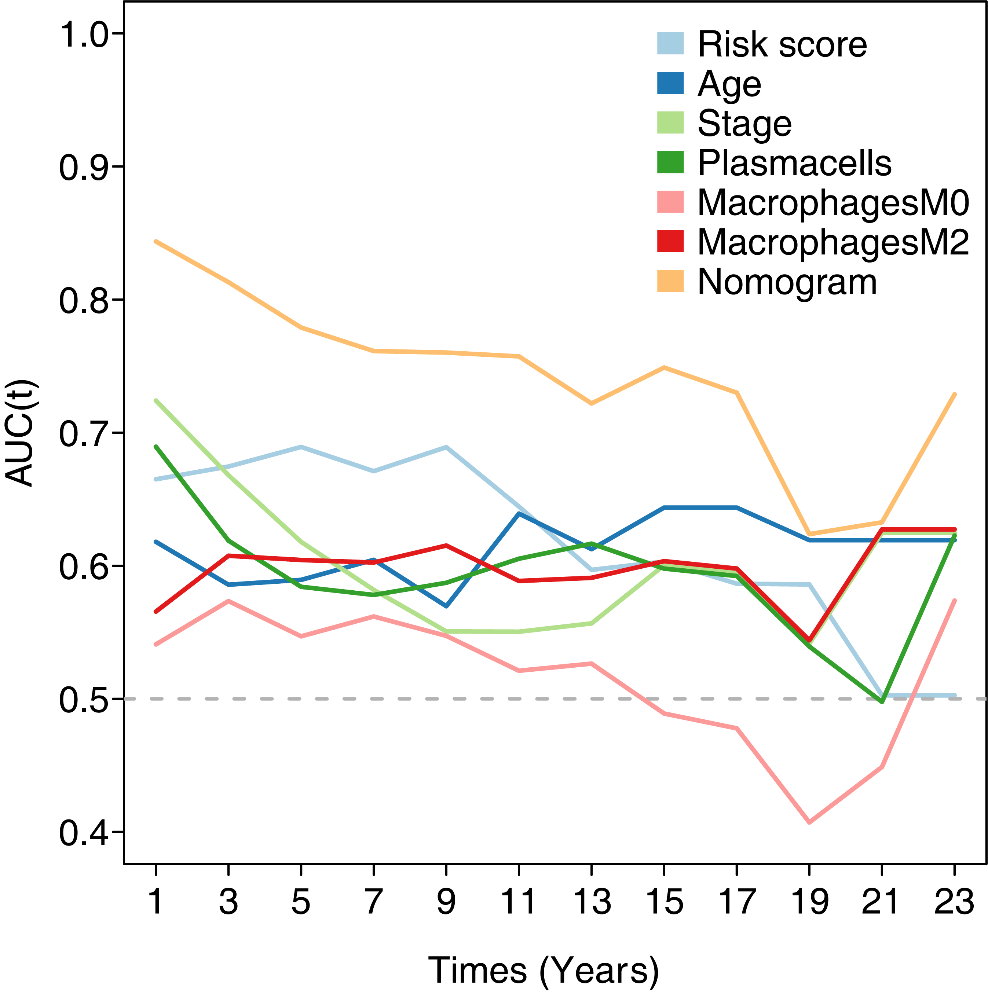


The comparison of predictive AUCs among the nomogram model and each factors.

**Fig. S8.** GO and KEGG functional enrichment analyses between two groups according to the nomogram score in TCGA.


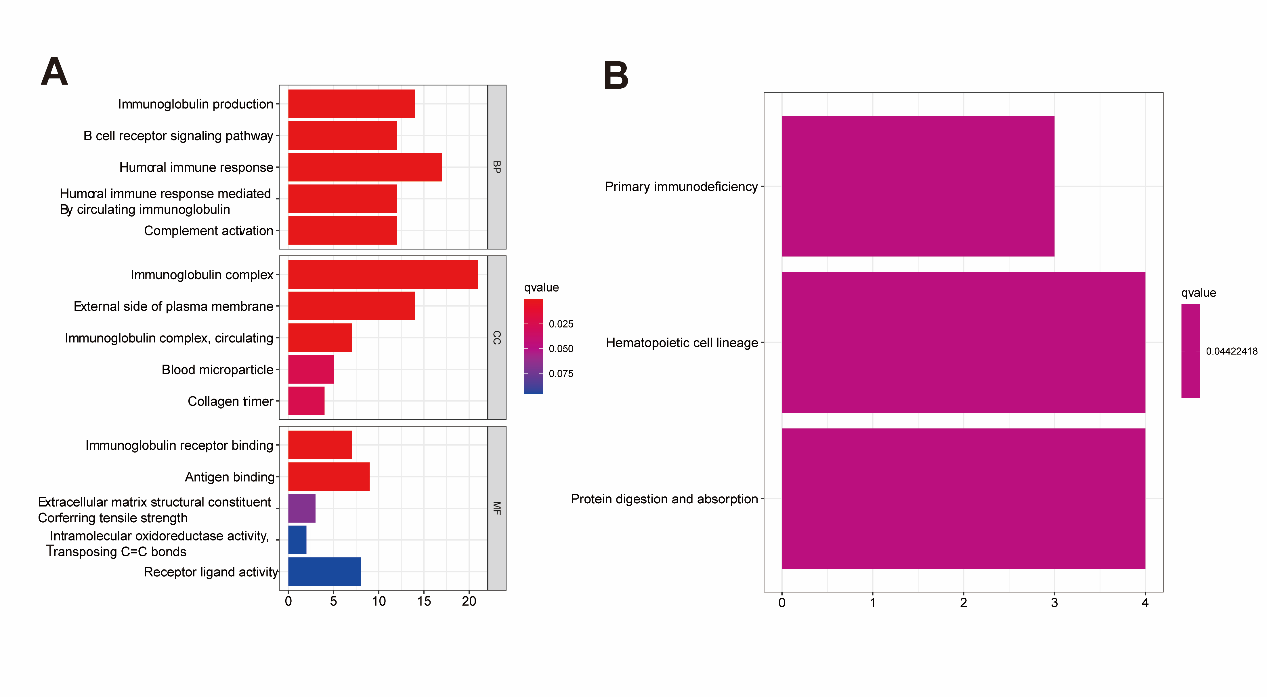


(A) GO enrichment of the DEGs between the high- and low-risk patients divided by nomogram score in the TCGA cohort.

(B) KEGG pathways of the DEGs between the high- and low-risk patients divided by nomogram score in the TCGA cohort.
